# A novel radiological classification system for cerebral gliomas: The Brain-Grid

**DOI:** 10.1371/journal.pone.0211243

**Published:** 2019-01-24

**Authors:** Francesco Latini, Markus Fahlström, Shala G. Berntsson, Elna-Marie Larsson, Anja Smits, Mats Ryttlefors

**Affiliations:** 1 Department of Neuroscience, Neurosurgery, Uppsala University, Uppsala, Sweden; 2 Department of Surgical Sciences, Radiology, Uppsala University, Uppsala, Sweden; 3 Department of Neuroscience, Neurology, Uppsala University, Uppsala, Sweden; 4 Institute of Neuroscience and Physiology, Department of Clinical Neuroscience, Sahlgrenska Academy, University of Gothenburg, Gothenburg, Sweden; University of Pécs Medical School, HUNGARY

## Abstract

**Purpose:**

Standard radiological/topographical classifications of gliomas often do not reflect the real extension of the tumor within the lobar-cortical anatomy. Furthermore, these systems do not provide information on the relationship between tumor growth and the subcortical white matter architecture. We propose the use of an anatomically standardized grid system (the Brain-Grid) to merge serial morphological magnetic resonance imaging (MRI) scans with a representative tractographic atlas. Two illustrative cases are presented to show the potential advantages of this classification system.

**Methods:**

MRI scans of 39 patients (WHO grade II and III gliomas) were analyzed with a standardized grid created by intersecting longitudinal lines on the axial, sagittal, and coronal planes. The anatomical landmarks were chosen from an average brain, spatially normalized to the Montreal Neurological Institute (MNI) space and the Talairach space. Major white matter pathways were reconstructed with a deterministic tracking algorithm on a reference atlas and analyzed using the Brain-Grid system.

**Results:**

In all, 48 brain grid voxels (areas defined by 3 coordinates, axial (A), coronal (C), sagittal (S) and numbers from 1 to 4) were delineated in each MRI sequence and on the tractographic atlas. The number of grid voxels infiltrated was consistent, also in the MNI space. The sub-cortical insula/basal ganglia (A3-C2-S2) and the fronto-insular region (A3-C2-S1) were most frequently involved. The inferior fronto-occipital fasciculus, anterior thalamic radiation, uncinate fasciculus, and external capsule were the most frequently associated pathways in both hemispheres.

**Conclusions:**

The Brain-Grid based classification system provides an accurate observational tool in all patients with suspected gliomas, based on the comparison of grid voxels on a morphological MRI and segmented white matter atlas. Important biological information on tumor kinetics including extension, speed, and preferential direction of progression can be observed and even predicted with this system. This novel classification can easily be applied to both prospective and retrospective cohorts of patients and increase our comprehension of glioma behavior.

## Introduction

Gliomas comprise approximately 30% of all primary CNS tumors and 80% of malignant brain tumors [1, 2]. Low-grade gliomas are WHO grade II tumors and are characterized by slow growth but extensive infiltration. They occur mainly in adult life, with a peak incidence around 30–40 years. The clinical course of low-grade gliomas is diverse, but all tumors transform into high-grade gliomas and will eventually lead to death [3].

From a clinical as well as tumor biological prospective, gliomas are extremely heterogeneous tumors. and an accurate classification system is mandatory to predict tumor behavior and to tailor individual treatment strategies. The differences in growth rate, the complex growth patterns and the resilience of gliomas suggest that tumor shape is a result of their kinetic features, reflecting the complex dynamic progression over time in interaction with the surrounding brain [4,5]. Although the patterns of brain invasion may be different for tumor types of different lineage origin, invasiveness seems to be an intrinsic property of all glial tumors [4, 6]. Bio-mathematical models have been used to predict tumor shape based on 2 main phenomena involved in glioma progression: proliferation and infiltration [5,7–11]. When proliferation is the predominant phenomenon, the effects of tumor infiltration do not affect tumor shape, which is grossly bulky, whereas diffusively infiltrating tumors with low proliferation will lead to a complex shape with digitations along the deep white matter fibers [5,8,10,12–14]. Differences in cortical subcortical infiltration/dislocation patterns are related to the histological origin and may depend on permissive nature of neighboring structures around the tumor. Dissemination of astrocytic tumors seems to be confined to the white matter near the cortex or deep gray nuclei, which, under certain circumstances, act as barriers to the invasion of some gliomas [6,15,16]. This phenomenon is less prominent in oligodendrogliomas, which will frequently invade the cortex and are less likely to respect anatomic boundaries [17]. On the other hand, tumor grade does not seem to be strictly related to the degree of local invasion, and low-grade astrocytomas (WHO II) may show extensive infiltration of adjacent subcortical regions [18].

The standard radiological classification of gliomas is still based on "local" neuro-radiological anatomic features [19–22]. It is common practice to describe a glioma based on the nomenclature of major lobes invaded despite the subcortical extension. For instance, a frontally located glioma can often invade the basal ganglia or even the midline through the corpus callosum, but is still classified as a frontal glioma [19–22]. Additional classification systems for low-grade gliomas use tumor topography, based on frequent tumor locations, such as limbic and paralimbic structures, the insula, and supplementary motor area [20,23–25], or on probabilistic location maps derived from retrospective analyses [26]. For bulky tumors, it seems reasonable to describe the location by naming the affected lobe, as is usually done [19–22]. However, as infiltration occurs preferentially along white matter fibers, the white matter architecture needs to be incorporated into the radiological classification.

Important advances in the non-invasive diagnosis of tumor infiltration/dislocation of white matter tracts have been made by tractographic studies [13,23,27–31], but this technique has never been incorporated into a classification system. This may be due to a still open debate about the technical limitations encountered by tractography (operator-dependent variables, dependency on scanning device use, algorithm problems, peritumoral tissue abnormalities, post-radiation white matter abnormalities) [30,32–36]. The use of diffusion tractography imaging (DTI) atlases, which represented a milestone for the localization of the white matter bundles in both physiological and pathological conditions [37–41], seems limited in daily clinical practice due to at least 2 reasons: the necessity to analyze MRI sequences in a normalized space and the lack of landmarks in the deep white matter for longitudinal comparison in time. Moreover, even patients in whom it has not been possible to perform tractography, white matter architecture and glioma progression should be analyzed.

In this study, we propose a grid system (the Brain-Grid) based on a fixed number of lines across anatomical landmarks (deep and superficial) to divide the entire brain into comparable voxels units (grid voxels). In addition, inherent normal white matter architecture, incorporating major commissural, associative, and projection pathway fiber tracts, was defined by tractography using average brain data from the Human Connectome Project (HCP). Combining the Brain-Grid system and white matter architecture, we developed a novel tool merging local radiological anatomy, white matter architecture, morphology, and kinetics of gliomas in one classification system. To illustrate the advantages of the Brain-Grid classification system, we presented the temporal course of 2 patients who were conservatively managed following radiological diagnosis. We show that this easily applicable non-invasive classification system provides new information on cortico-subcortical glioma localization, including a prediction of the specific white matter structures associated with tumor progression.

## Methods and materials

### Patient population

Thirty-nine patients (>18 years) presenting with a radiological diagnosis of suspected low-grade glioma were consecutively recruited at the Department of Neurosurgery, Uppsala University-Hospital, Uppsala, Sweden, and enrolled in the study between February 2010 and September 2015, as previously described [42]. The study was approved by the local ethics committee, and written informed consent was obtained from all patients prior to participation. The capacity to consent was ascertained through clinical and mental status evaluation at the time for inclusion in the study. Inclusion criteria were morphological MRI findings with high signal intensity on T2-weighted fluid attenuated inversion recovery (FLAIR) sequence and a 3D T1-weighted sequence with no or minimal (patchy and faint) contrast enhancement suggestive of a low-grade glioma. All MR images were re-evaluated by the first author of this study and reclassified according to the Brain-Grid system. If additional MRI scans had been performed earlier, these scans were included in the analysis.

The quality of the images, registration and fusion within MNI space was attested by a MRI research engineer (MF). The Brain-Grid technique was reproduced by two neurosurgeons experienced in neuroimaging (FL and MR) and a Professor in Neuroradiology (EML). The final voxel count and the analysis of the results was performed by a neurosurgeon experienced in neuroimaging (FL).

### The anatomical definition of the Brain-Grid classification system

**Fig 1:**
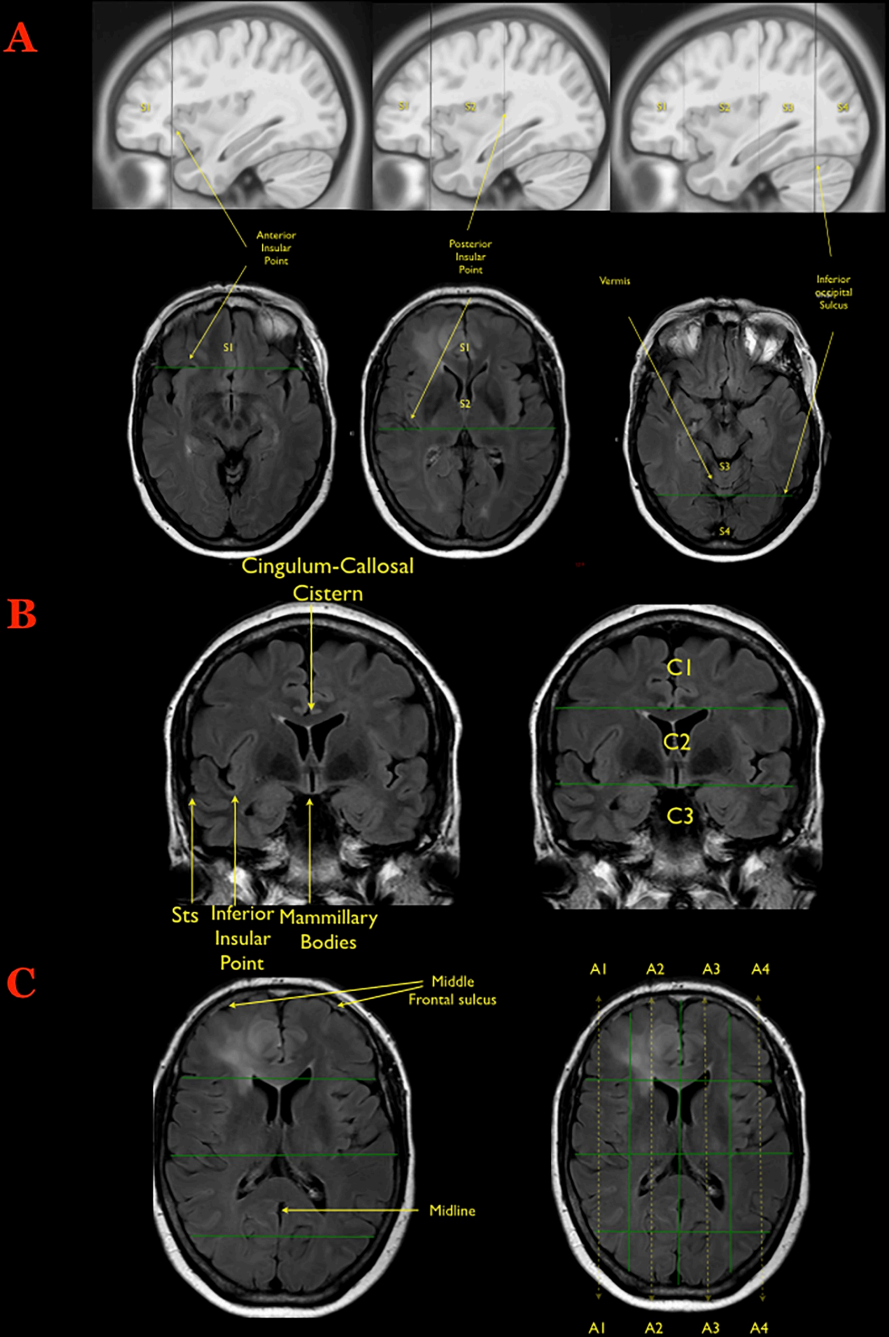
Creation of the Brain-Grid. **A)** The first step during the creation of the Brain-Grid is illustrated on the sagittal or axial slices: The anterior insular point (the most anterior landmark of the insular sulcus) is identified on both sides and the first line is drawn on the sagittal plane (MNI: Y 28, Talairach: Y25). The second line (drawn as well on the sagittal plane) is parallel to the previous one, crossing the posterior insular point (MNI: Y-23; Talairach: Y-24). On the midline (with sagittal view) the point where the calcarine fissure (V1) meets the most anterior portion of the parieto-occipital sulcus should be identified in order to track the third parallel line on the sagittal plane (MNI: Y-68; Talairach: Y-66). The same line crosses the temporo-occipital junction between the posterior portion of the fusiform gyrus and the inferior occipital sulcus more basally on the axial plane. The 3 lines on the sagittal plane will segment the whole brain into 4 grid voxels. The S1 voxel is the pre-insular/prefrontal portion of both hemispheres. The S2 is enclosed within the anterior insular point and posterior insular point (landmark for the second sagittal line). The S3 includes the retro-insular region and the parietal lobe, and the S4 includes primarily the occipital lobe and the border with the parieto-occipital sulcus. **B)** The second step during the creation of the Brain-Grid system is the identification of the right slice on the coronal plane (Into the MNI space: Y-5; Talairach Y -7). The first of the 2 parallel lines crosses the inferior insular point (the lowest limit of the insular sulcus) and the floor of the third ventricle that leads to the rounded shape of the mammillary bodies. In most patients, this horizontal line usually crosses the superior temporal sulcus on both sides (MNI: X0, Y-5, Z-13; Talairach: X0, Y-7, Z-7). The second line passes through the cistern/space between the cingular gyrus and the callosal body in the midline (MNI:X0, Y-5, Z33; Talairach: X0, Y-4, Z 31). **C)** Third step: Once the coronal segments and the sagittal segments are created, one should identify the middle frontal sulcus bilaterally, which is easily recognizable on the axial slice that shows the level of the lateral ventricle on the coronal reference (shown on the side). The 2 lines should be parallel to the midline, connecting this sulcus with the middle occipital gyrus crossing the white matter of the external capsule without invading the periventricular ependyma (right line, MNI: X33; Talairach: X32. left line, MNI:X-33; Talairach: X-32). The third and last line follows the midline along the falx and/or the septum pellucidum (MNI: X0; Talairach: X0). In this way 4 longitudinal segments are created, termed A1 to A4, from the right lateral side to the left lateral side.

**Fig 2:**
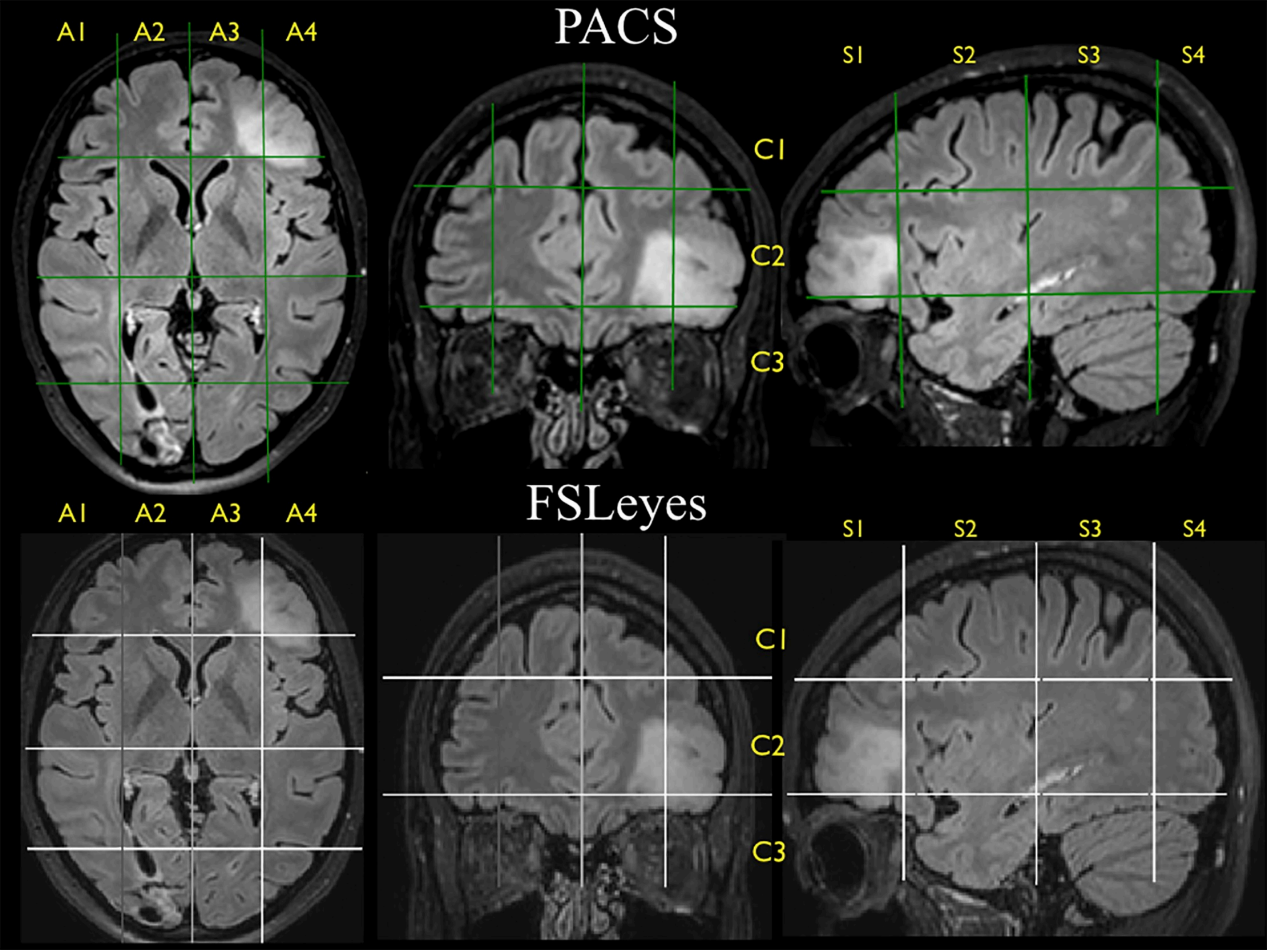
Brain-Grid voxels. Example of how each segment can be identified according to the lines drawn on the 3 axes within the PACS clinical image client (upper part) and within the MNI space using FSLeyes (lower part). The tumor seems to invade the lateral left axial segment, thus A4, but even the second medial segment is invaded, involving A3. Two coronal units are involved in the more anterior part of the grid (C2-C3). According to the sagittal subdivision, the second and third segments are invaded by the hyperintensity but only in the more lateral quadrants. The results on this slice are A3C2S1 + A3C3S1, plus A4C1S1, A4C1S2, A4C3S1, a total of 5 units.

The underlying anatomical landmarks defining the Brain-Grid system were defined based on a T1-weighted average brain in the MNI space. The MNI space coordinates were converted to Talairach coordinates using the MNI to Talairach coordinate converter (Yale BioImage Suite Software, www.bioimagesuite.org [43]. The anatomical landmarks used for the individual placement of each line comprising the Brain-Grid are shown in Fig 1. Furthermore, all lines were applied to cover the whole brain, thus making the Brain-Grid visible in each individual slice. Lines are defined in all 3 anatomical planes (axial, coronal, and sagittal). The Brain-Grid consist of 3 axial lines, 2 coronal lines, and 3 sagittal lines, whereas the intersection of these lines creates 48 grid voxels. Each voxel can be identified using simple nomenclature. First, radiological orientation is considered. In the axial (A) plane, voxels are labeled 1–4, right to left direction. In the coronal (C) plane, voxels are labeled 1–3, cranio-caudal direction. In the sagittal (S), voxels are labeled 1–4, anterior-posterior direction. A stepwise instruction describing the placement of the Brain-Grid classification system can be found in Fig 1. The labeling used in the Brain-Grid classification system is graphically presented in Fig 2.

### Brain-Grid classification system in patients

The application of the Brain-Grid classification system in patients was performed using T2 turbo spin echo (TSE) or T2-FLAIR images in the Vue picture archiving and communication system (PACS) software (version 11.1.0, Carestream Health Inc., Rochester, NY, USA.). Multiplanar reconstructions were performed to orient the image in relation to the anterior commissure–posterior commissure line. The Brain-Grid classification system was then applied as described above. In the case of infiltration/dislocation of the anatomical landmarks, the contralateral landmark was identified in order to track the grid lines. Vue PACS was used to segment the lesions with the aid of a semiautomatic method (livewire algorithm). The total tumor volume was calculated on T2-FLAIR or T2-TSE images using the technique previously described by our group [44–45]. The number of grid voxels involved in the tumor extension was registered based on the defined tumor lesion. Registered grid voxels were also compared to the white matter architecture, defining fiber tracts involved by tumor extension. The distance between the grid line and the visible tumor border was recorded and compared in patients with multiple MRI scans during the follow-up period. Furthermore, T2-TSE or T2-FLAIR images of 10 randomly chosen patients were spatially normalized to the MNI space using the SPM12 toolbox (Wellcome Trust Center for Neuroimaging, London, UK). Applying the Brain-Grid classification system using the FSLeyes software (https://fsl.fmrib.ox.ac.uk/fsl/fslwiki/FSLeyes) in the space, the number of voxels involved in the tumor was counted and compared to a corresponding value from the Vue PACS in the patient-specific space (Fig 2).

### The human connectome project 488

The WU-Minn HCP consortium is an institutional, review board-approved, NIH-funded project led by Washington University, University of Minnesota, and Oxford University [46]. The HCP-488 standard template is based on diffusion scanning data acquired from 488 healthy subjects (199 males, 289 females, average age 29.15 years, standard deviation 3.47 years). The diffusion data were acquired using a Siemens 3.0 T Skyra scanner with a 2-dimensional spin-echo single-shot multiband echo planar imaging sequence, a multiband factor of 3 and monopolar gradient pulse. The spatial resolution was 1.25 mm isotropic; repetition time was 5500 milliseconds; and echo time was 89 milliseconds. A multishell diffusion scheme was used; b values were 1000, 2000, and 3000 s/mm2. The total number of diffusion sampling directions was 270. The total scanning time was approximately 55 minutes. A detailed description of data acquisition and processing, on behalf of the WU-Minn HCP Consortium, can be found in Sotiropoulos et al. [47]. The diffusion data were reconstructed in the MNI space using q-space diffeomorphic reconstruction [48]. A standard template, the HCP-488 atlas, was created averaging the reconstructed data of the 488 subjects (DSI Studio, http://dsi-studio.labsolver.org/download-images). A deterministic fiber tracking algorithm was used for whole brain fiber tracking [49,50].

### HCP-488 template-based white matter architecture

The inherent normal white matter architecture incorporated in the Brain-Grid classification system was defined by HCP-488 template-based fiber tracking including 34 major white matter bundles/structures. We applied a knowledge-based multiple region-of-interest (ROI) approach in which the tracking algorithm was initiated from user-defined seed regions. The anatomical placement of the ROIs was selected by using the most validated DTI atlases [37–39,41] as references. ROI placement and interpretations of tract location and trajectory were furthermore assisted by anatomical T1-weighted average brain and modern neuroanatomical references works [29,51,52]. A single-ROI approach was used for the anterior commissure (AC), corpus callosum (CC), internal capsule (IC), external capsule (ExC), and fornix (Fo). A 2-ROI approach was used for the cingulum, inferior longitudinal fasciculus (ILF), middle longitudinal fasciculus (MLF), inferior fronto-occipital fasciculus (IFOF), uncinate (UF), frontal aslant tract (FAT), cortico-spinal tract (CST), optic radiations (OR), anterior thalamic radiation (ATR), and vertical occipital fasciculus (VOF). A 2-ROI approach was also used to separate the 2 segments of the superior longitudinal fasciculus (SLF) and arcuate fasciculus (AF) (see S1 and S2 Appendices for functional references for each tract or structure).

**Fig 3:**
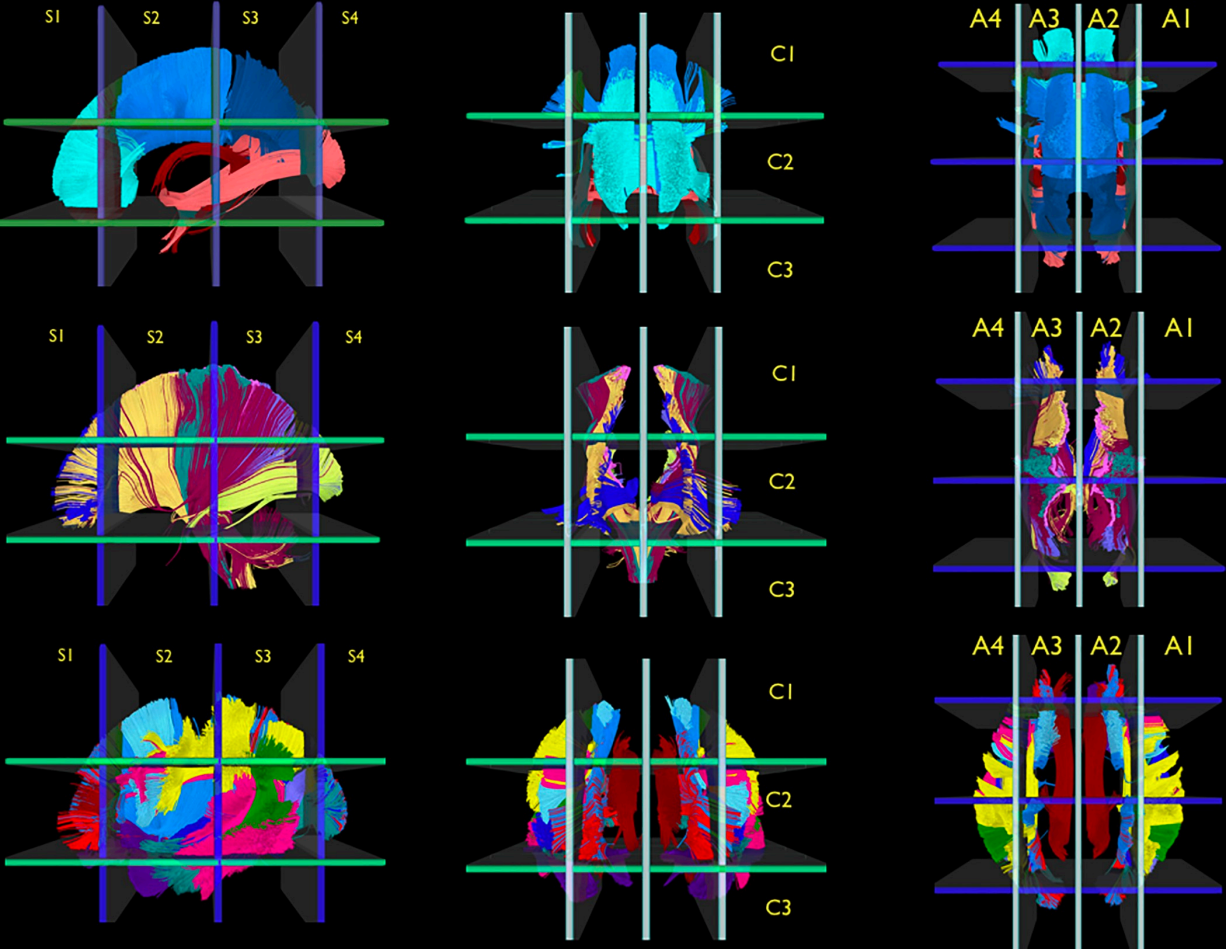
Reference white matter atlas. The illustration shows the reference white matter atlas reconstructed from the HCP-template and analyzed with the Brain-Grid system. The major commissural (CC, AC, and Fo, first row), projection pathways (CST, IC, OR, TR, second row), and associative pathways (cingulum, SLF components, VOF, ILF, MLF, IFOF, UF, and FAT, third row) of both hemispheres are displayed in a left sagittal view (first column), anterior coronal view (second column), and superior-axial view (third column). The white matter bundles were tracked from the HCP-488 template within the MNI space and then analyzed using the Brain-Grid system with the number and letters for the grid voxels displayed for each projection. (See main text and the S1 and S2 Appendices for details on the single white matter bundles).

The derived white matter architecture (all fiber tracts and white matter structures) was incorporated into the Brain-Grid system as seen in Fig 3 and S1 and S2 Appendices.

## Results

### Patients and tumor characteristics

The clinical, radiological, and tumor characteristics of the patients are shown in Table 1. The mean age of the patients was 46.9 years. Seven patients (18%) underwent a diagnostic biopsy, while 32 patients (82%) were treated with surgical resection. Three patients (all included early in 2010) were initially followed with a watch-and-wait strategy before undergoing surgery (1 biopsy, 2 resection), reflecting the surgical strategy at our clinic during the initial recruitment period [21]. The histopathological diagnoses were 19 astrocytomas (12 grade II and 7 grade III) and 20 oligodendrogliomas (12 grade II and 8 grade III) [53]. The Brain-Grid classification system was applied in 105 MRI scans (see Figs 1 and 2 for instructions and S1 and S2 Appendices).

**Table 1:** Summary of the clinical, histological, and radiological information on each patient enrolled in this study. M: Male; F: Female; Histo: histology; O: Oligodendroglioma; A: Astrocytoma; R: right; L: Left; P: Parietal; F: Frontal; T: Temporal; O: Occipital; I: Insular; D: Diffuse; B: Bulky; PAR: Partial seizure; GEN: Generalized seizure; ICH: Intracerebral Hematoma; R: Surgical resection; B: Biopsy.

*P* n°	Gender	AGE	Histo	WHO Grade	SIDE	Local Radiological Anatomy	Diffuse/ Bulky	Volume (cm3)	BG- voxels	Onset	Biopsy/ Resection	MRI n°
**1**	M	61	A	2	R	P	B	24.4	4	INCIDENTAL/TINNITUS	R	2
**2**	M	66	A	2	L	F	B	31.1	7	SEIZURE PAR-COMPLEX	B	3
**3**	F	65	A	3	B	F	D	108	16	SEIZURES PAR	B	4
**4**	F	30	O	2	R	F	B	5.1	1	HEADACHE	R	4
**5**	M	38	A	2	L	F	B	33	2	SEIZURES PAR	R	4
**6**	F	27	A	3	R	F	B	54.6	8	SEIZURE PAR	R	2
**7**	M	31	O	3	R	T	D	98.2	13	SEIZURE GEN	R	2
**8**	F	31	A	3	L	F-P	B	38.5	5	SEIZURE PAR	B	9
**9**	F	31	O	2	R	F	D	114.3	12	SEIZURE GEN	R	2
**10**	F	42	O	2	R	F	B	71.7	8	SEIZURE GEN	R	2
**11**	M	25	O	2	L	P	D	30.9	7	NUMBNESS R LEG	R	2
**12**	F	78	A	3	L	F	D	10.4	1	SEIZURE GEN	R	2
**13**	F	62	A	2	R	T-I	D	58.5	10	SEIZURE PAR	B	6
**14**	F	22	A	2	R	F-P	B	61.6	9	SEIZURE PAR	R	2
**15**	M	70	A	3	R	T	D	85.7	13	SEIZURE PAR	B	2
**16**	F	40	O	3	R	F-P	D	36.1	7	SEIZURE PAR	R	2
**17**	M	52	A	2	R	P-O	D	19.2	7	SEIZURE GEN	R	2
**18**	M	41	A	2	L	T	D	26.7	7	SEIZURE GEN	R	2
**19**	F	44	A	2	L	FTI	D	30.4	8	SEIZURE GEN	R	2
**20**	M	67	A	2	L	T-P	D	141.7	14	SEIZURE GEN	B	4
**21**	F	43	O	2	R	F	D	78.5	11	SEIZURE GEN	R	2
**22**	F	66	A	2	L	T	D	64.5	10	SEIZURE PAR	R	2
**23**	M	44	O	2	R	F	B	22.9	2	SEIZURE GEN	R	2
**24**	M	60	A	2	R	F	B	20.1	6	WEAKNESS L HEMIBODY	R	2
**25**	M	54	O	2	L	T	B	33.9	4	SEIZURE PAR	R	2
**26**	M	44	O	2	R	F	B	37.2	4	SEIZURE GEN	R	2
**27**	F	40	O	2	L	F	D	17.2	6	HEADACHE/ DYZZINESS	R	4
**28**	F	68	O	3	L	F	B	6.5	1	SEIZURE PAR	R	2
**29**	M	34	O	2	L	F-I	D	35.1	6	HEADACHE	B	7
**30**	M	26	O	2	R	F	B	36.7	6	SEIZURE GEN	R	2
**31**	M	39	O	2	L	F	D	62.1	6	HEADACHE	R	2
**32**	F	50	A	3	L	T	D	129.8	12	SEIZURE GEN	R	2
**33**	M	54	O	3	L	F-T-I	D	93.3	13	SEIZURE PAR-COMPLEX	R	2
**34**	F	61	A	3	R	F	D	34.3	7	SEIZURE GEN	R	2
**35**	M	53	O	3	L	F	D	91.1	12	SEIZURE GEN	R	2
**36**	M	26	O	3	L	F	D	34.1	3	INCIDENTAL	R	2
**37**	F	45	O	3	L	F	B	24.6	5	ICH /VISION IMPAIRMENT	R	2
**38**	M	47	O	3	R	F	D	65.5	8	SEIZURE GEN	R	2
**39**	M	33	A	2	L	F	B	50.6	5	SEIZURE GEN	R	2

### Radiological characteristics

According to the standard topographic radiological classification, the tumor was located in the frontal lobe in 25 patients (64%), temporal (18%) lobe or parietal lobe (18%) in 7 patients each. In 20 cases the tumor was detected on the left side, 18 involved the right hemisphere, and in 1 case the extension of the lesion was bilateral. The mean tumor volume was 54.4 cm^3^ (range 5.5–141.7 cm^3^). The tumors were considered bulky, with well-defined margins (without any finger-like hyperintense signals on T2 or T2 FLAIR sequences) in 16 cases (Table 1) [14]. Among the bulky cases, 12 tumors were WHO grade II (8 oligo and 4 astro) and 4 tumors were WHO grade III (3 oligo and 1 astro). Diffuse tumor margins with unclear, and irregular signal intensity on T2-FLAIR sequences were found in 23 cases [14]. Among these 23 diffuse cases, 12 tumors were WHO grade II (7 oligo and 5 astro) and 11 tumors were WHO grade III (7 oligo and 4 astro) (Table 1).

### Brain-Grid application in patients with gliomas

The Brain-Grid classification system could be applied in all 39 patients and all MRI sequences. No limitations with regard to identifying the anatomical landmarks were encountered. We analyzed findings according to the quantitative and qualitative involvement of grid voxels, considering tumor location, white matter architecture, and longitudinal examinations.

**Fig 4:**
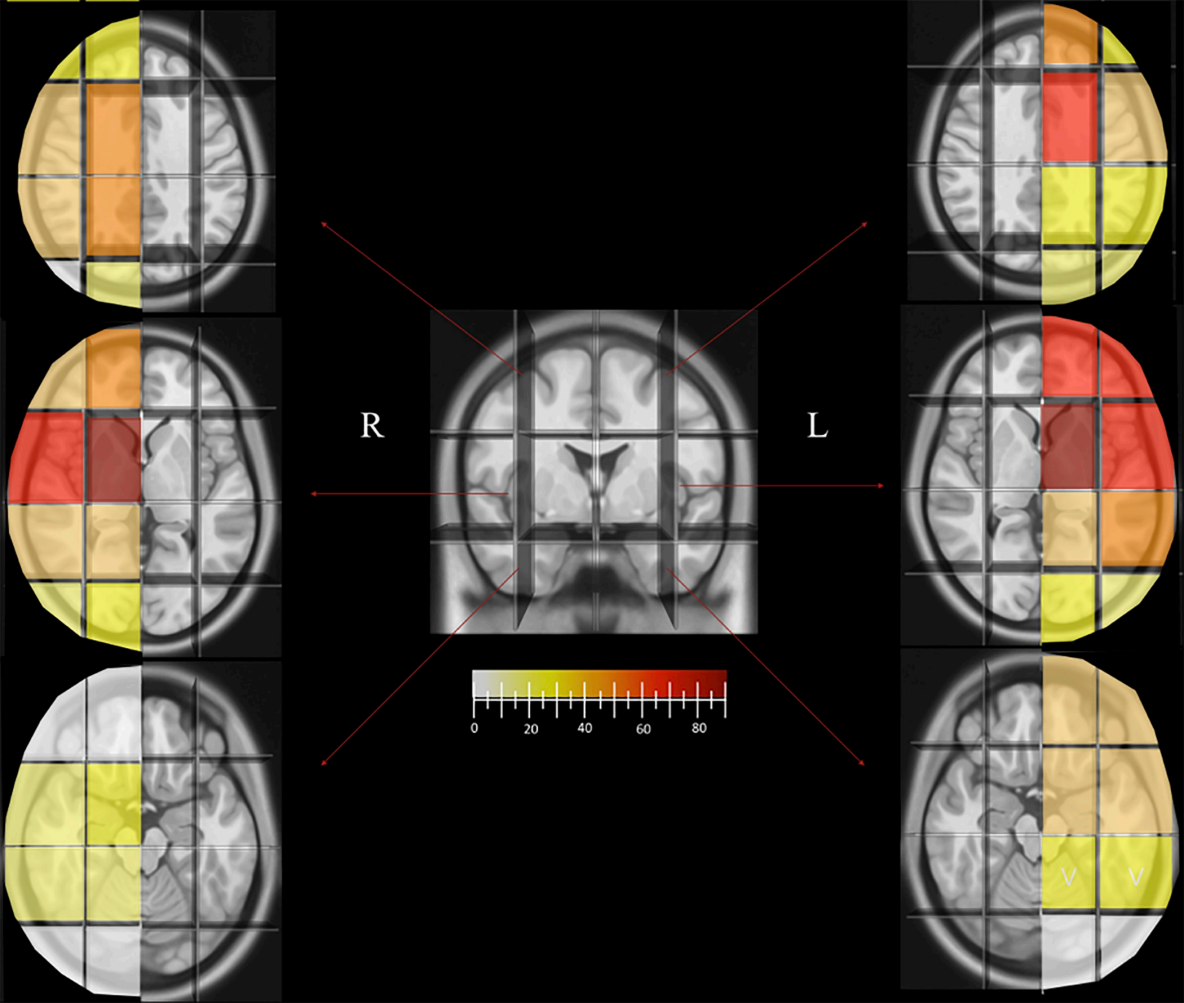
Brain-Grid voxels frequency. The picture shows the trend in the frequency of Brain-Grid voxels in patients with left- or right-sided tumors according to a color gradient from white (0–5%) to dark red (more than 80% of the cases). The cut-off for a high frequency (intense orange) was set at 50% of the lesions. The A1C2S2 and A2C2S2 are most frequently involved on the right side, while A3C1S2, A3C2S2, and A4C2S2 are most frequently involved in patients with left-sided tumors, with the highest incidence being displayed by the median and central voxels bilaterally (subcortical insular regions-basal ganglia), both involved in over 80% of the cases (dark red).

The mean number of grid voxels that were considered to be infiltrated by the tumor in patients at the time of recruitment was 7.4 (range 1–16). The average number grid voxels involved in bulky tumors (16/39) was 5.1, whereas in diffuse gliomas (23/39) the average number was 9. The mean number of these units involved in grade II tumors was 6.6 (range 2–14) and 8 in WHO grade III tumors (range 1–16). Analysis of the incidence of single grid voxels showed that 52.7% (152/288) of the infiltrated grid voxels were on the left side. The A3-C2-S2 and the A3-C2-S1 (corresponding respectively to the subcortical insula/external capsule/basal ganglia and the frontoinsular region) were the most frequently infiltrated, in 86% (18/21) and 66% (14/21) of the cases with left or bilateral infiltration. In patients with right-sided or bilateral infiltration, the A2-C2-S2 (89%, 17/19) and the A1-C2-S2 (63%, 12/19) were most frequently involved. The number and location of involved Brain-Grid voxels was consistent in all the cases re-analyzed into the MNI space (Fig 2). The frequency of tumor involvement for each voxel is displayed in Fig 4. The analysis of the voxel infiltration in serial MRI investigations was possible in 9 patients using multiple MRIs (See Table 1 and the 2 illustrative cases below). No relevant difference was recorded between the initial MRI and a second MRI taken within 3 months from the first one in those patients in whom a second MRI was performed.

### Brain-Grid application on the HCP-488 template-based white matter atlas

According to the general organization of white matter architecture [54], the major commissural (CC, AC, Fo), projection pathways (CST, IC, OR, TR; and external capsule, ExC) and associative pathways/structures (Ci, SLF, VOF, ILF, MLF, IFOF, UF, and FAT) of both hemispheres were 3-dimensionally reconstructed within the MNI space. The anatomical results reproduced the main distribution of each bundle within the average space as described by other authors [37–41].

The derived white matter architecture (all fiber tracts and white matter structures) was incorporated into the Brain-Grid atlas, as seen in Fig 3 and S1 and S2 Appendices.

The qualitative analysis of the grid voxels based on the HCP-488 template-based white matter architecture (Fig 3 and S1 and S2 Appendices) showed that the IFOF, UF, external capsule, and ATR were the white matter structures most frequently associated with the tumors in sub-insular/basal ganglia voxels (A2-C2-S2 and A3-C2-S2). The ATR, the genu of CC, cingulum, and anterior portion of IFOF were the most often identified tracts in tumors harboring the fronto-medial voxels (A2-C2-S1 and A3-C2-S1). The AF, hSLF, and FAT were second in frequency in association with insular cortical /fronto-opercular region (A1-C2-S2) on the right side.

### Illustrative cases

Here we describe 2 illustrative patients, both in their early thirties at the time of radiological diagnosis, who, for various reasons, were not offered surgical resection during the early course of their disease.

#### Case 1 (Fig 5)

This patient underwent an MRI scan of the brain in 2002 because of headache. A 1.8 cm^3^ hyperintense lesion on T2FLAIR sequences in the right medial frontal lobe was found. The patient was initially managed conservatively. Retrospective volumetric segmentation of the tumor, performed as part of the present study, revealed a continuous increase in volume over time, from 1.8 cm^3^ early 2002, to 2.4 cm^3^ late 2002, to 5.3 cm^3^ in 2004. Brain-Grid analysis in 2002 showed the involvement of only 1 grid voxel (A2-C2-S1) harboring the short-intermediate fibers medially and anteriorly with respect to the callosal fibers of the genu of the CC, in close relationship with the lower portion of the cingulum fibers. An MRI scan in 2010 showed a dramatic increase in volume (36.1 cm^3^) and the patient was scheduled for surgery. The Brain-Grid analysis revealed involvement of 7 grid voxels in 2010, with invasion of the A3-C2-S1 segment through the callosal fibers beyond the midline, but also in the cranio-caudal direction toward the C1 and C3 voxels following the cingulum fibers, the frontal longitudinal pathways such as anterior thalamic radiation and the frontal portion of the IFOF more laterally. Surgery was carried out in 2011, and histological diagnosis revealed oligodendroglioma WHO grade II.

**Fig 5:**
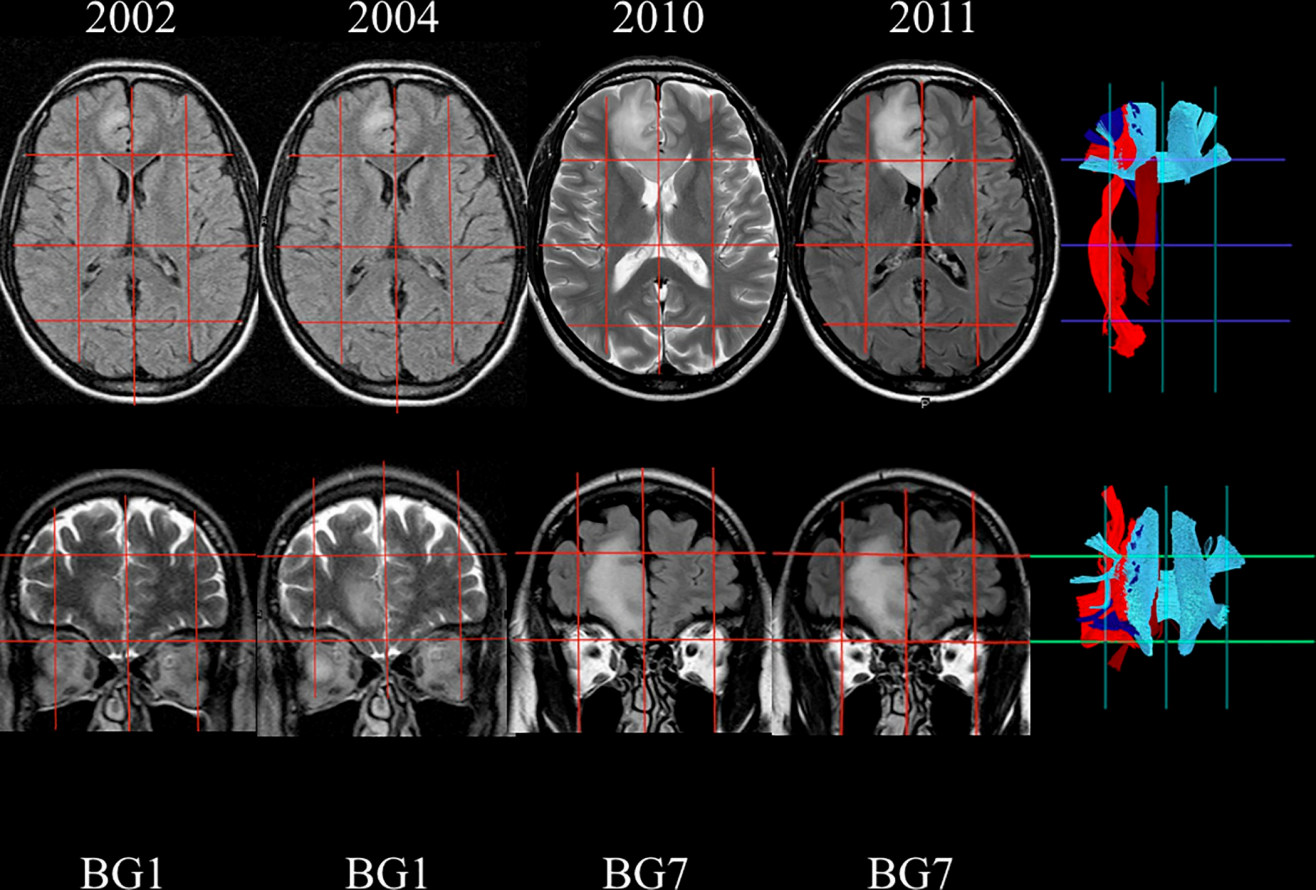
Radiological follow-up of Illustrative case #1. The figure illustrates the way in which the Brain-Grid system can add some important information about the white matter bundles infiltrated during a longitudinal follow-up. Axial and coronal slices show tumor evolution on morphological T2/ FLAIR MR sequences from 2002 and onwards. The signal hyperintensity evolved from 1 BG voxel to 7 BG voxels in both the coronal and axial direction. On the right side, the BG Atlas was used as a reference for the tractographic reconstructions, summarizing the major white matter bundles involved during the tumor progression. The genu of the corpus callosum (light blue), IFOF (red), cingulum (dark red), anterior thalamic radiation (dark blue).

#### Case 2 (Fig 6)

In 2010, this patient underwent an MRI scan of the brain because of chronic headache. The MRI showed a hyperintense bulky lesion without contrast enhancement in the left insula. Serial MRI scans revealed a rapid increase in tumor volume (84.8 cm^3^ in 2013). A diagnostic biopsy was performed, and histological examination demonstrated an oligodendroglioma WHO grade II. The decided treatment strategy was radiotherapy without surgery. After an initial decrease in tumor volume (from 48.7 cm^3^ in 2014 to 43.2 cm^3^ in 2015), the patient had a generalized seizure, and in 2016 the MRI scan showed an increase in tumor volume to 58.2 cm^3^. An MRI scan late in 2016 revealed a dramatic diffuse infiltration of the tumor to the contralateral hemisphere, basal ganglia region, and brainstem.

**Fig 6:**
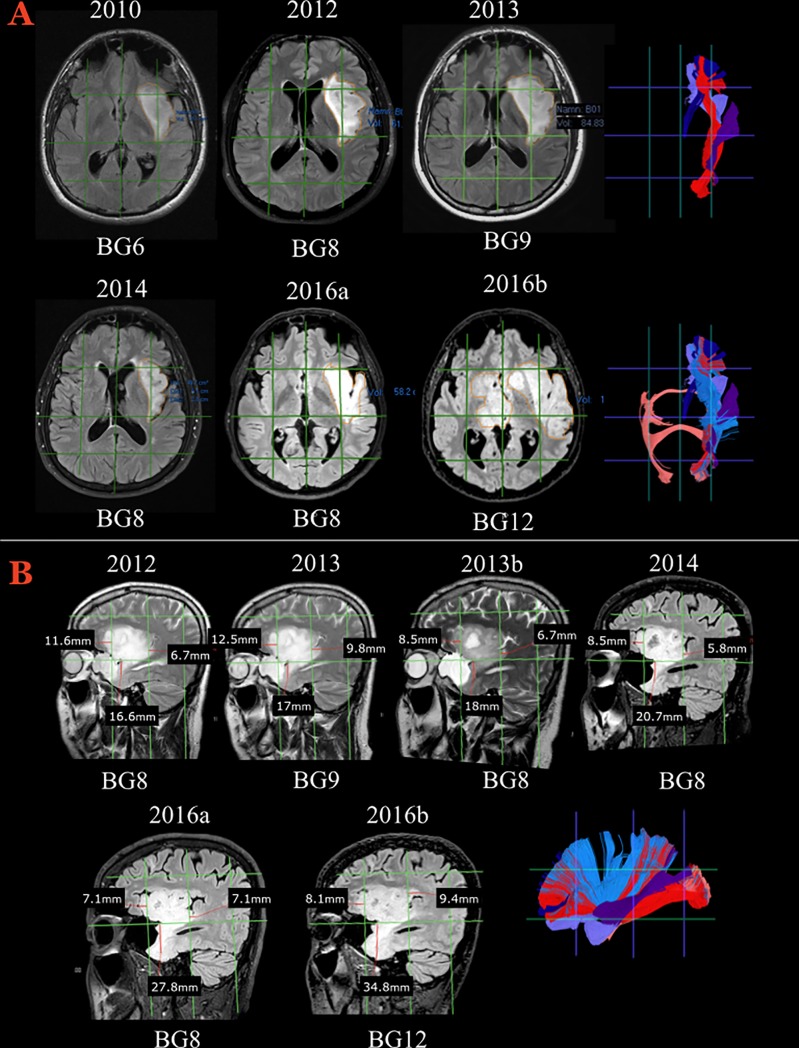
The radiological course of Illustrative case #2. **A)** Axial slices showing morphological FLAIR MR sequences during a longitudinal follow-up from 2010 and onwards. The signal hyperintensity evolved from 6 Brain-Grid units to 12 units, with a clear morphological transformation from a bulky shape to a diffuse and digitated shape infiltrating along the subcortical white matter. On the right, tractographic reconstructions from the Brain-Grid atlas revealed the major white matter involved during the progression. External capsule (light blue), UF (violet), IFOF (red), anterior thalamic radiation (dark blue), MLF (purple), AC (salmon). Follow-up MRIs in 2012 and 2013 demonstrated increased tumor volume involving also a radial extension of hyperintensity from the insula through the extreme and external capsule. Eight grid voxels were infiltrated in 2012, 9 voxels in 2013, with involvement of the S3 areas on both the lateral side (A4) and medial side (A3) within the intermediate coronal area (C2). The invasion at this time point, as shown in A–B, is more prominent through the posterior portion of the insula and sub-insular white matter. The potential pathways of infiltration are represented by the MLF fibers on the lateral (A4) grid voxel and the IFOF fibers medially (A3), caudally through the periventricular white matter (within the C3 area). After radiotherapy, the tumor volume as well as the infiltration along the longitudinal posterior pathways decreased significantly. The number of segments decreased to 8 due to a reduction of the hyperintensity in the A3C3S3 voxel. **B)** Details of the radiological follow-up between 2011 and 2016 that capture the switch from a bulky shape to a more diffuse and infiltrative appearance. On the right side, the Brain-Grid tractographic reconstructions summarizing the major white matter bundles involved during tumor progression. External capsule (light blue), UF (violet), IFOF (red), anterior thalamic radiation (dark blue), MLF (purple), AC (salmon). The sagittal projection shows that further infiltration along the antero-ventral pathways (UF) was not prevented by radiotherapy and that slow but continuous tumor growth occurred during the 3 years following radiotherapy. In 2016, when the entire anterior temporo-basal area was infiltrated, the number of infiltrated grid voxels was 12, showing also interhemispheric spread through the anterior commissure to the medial (A2) and intermediate coronal (C2) S2 and S3 grid voxels.

The Brain-Grid analysis identified the involvement of 6 grid voxels in 2010, at which time the tumor shape was bulky and restricted to the left frontoinsular region (A3-4-C2-S1-2). The extreme capsule and the ventral external capsule with the IFOF and UF were possibly already involved at radiological diagnosis. Eight grid voxels were infiltrated in 2012 and 9 voxels in 2013, with involvement of the S3 areas on both the lateral side (A4) and the medial side (A3) within the intermediate coronal area (C2). At this time, tumor invasion involved the MLF fibers in the lateral (A4) grid voxel and the IFOF fibers medially (A3) and caudally through the periventricular white matter (within the C3 area).

After radiotherapy, the tumor volume as well as the infiltration along the longitudinal posterior pathways decreased significantly. The number of segments decreased to 8 due to a reduction of the hyperintensity in the A3-C3-S3 voxel. However, further infiltration along the antero-ventral pathways (UF) was not prevented by radiotherapy, and slow but continuous progression occurred during 3 years following radiotherapy (Fig 6 B). In 2016, the number of infiltrated grid voxels was 12, showing also interhemispheric spread through the anterior commissure to the medial (A2) and intermediate coronal (C2) S2 and S3 grid voxels.

## Discussion

We present a novel, easily applicable Brain-Grid system for standardized radiological classification and longitudinal observation of intracerebral gliomas. Cortical anatomy, subcortical white matter bundles, and gliomas are integrated through an anatomical normalization of brain images.

The Brain-Grid classification system could consistently be applied in all morphological MRI sequences (a total of 105 MRI scans), regardless of the size, location, or radiological features of the tumors. The anatomical landmarks defining the Brain-Grid were easily visualized on standard clinical MR sequences, such as T2-TSE and T2-FLAIR. The number of grid voxels involved in the T2-FLAIR hyperintense signal was re-evaluated using a second-image software in the MNI space (Fig 2), confirming the consistency between patient-specific space evaluation and the MNI space, and thus supporting the use of anatomical landmarks in patient-specific MR images. This is necessary if the grid system is to be applied in daily clinical practice.

Observing tumor progression in serial MRIs through focus on brain grid voxels instead of lobar-radiological anatomy demonstrated some of the potential applications of this classification system. The first illustrative case confirmed that the standard classification systems fail to describe in spatial detail the natural evolution of a frontal tumor. Because it was classified as a frontal glioma at radiological diagnosis, important details were not noticed over the years due to the absence of subcortical landmarks. Even the constant tumor expansion demonstrated by standard volume computation [55,56] did not capture enough details about the possible involvement of the white matter bundles, details which were, however, provided by the grid voxel lines’ use of deep subcortical landmarks. In Illustrative case #1, the Brain-Grid classification system clearly showed the direction of tumor progression, laterally to the ATR and IFOF, medially through the genu of CC fibers, while in cranio-caudal direction through the cingulum fibers. With use of the Brain-Grid classification system, this information could be available in all the patients at the time of radiological diagnosis regardless of technical or medical limitations, even if a DTI is not available. Then, a tailored surgical strategy planned on the basis of a more extended resection of the closest white matter structures associated with tumor progression would potentially improve the neurosurgical/oncological result. The second case illustrates how changes in the direction of white matter infiltration after radiotherapy could be easily missed with use of standard classification systems. In this case, the tumor was first considered insular with temporal lobe involvement. However, the kinetics of this gliomas harboring 2 or 3 lobes with irregular subcortical extensions is very difficult to investigate without deep anatomical landmarks. On re-analysis using the sovra-impression of the Brain-Grid classification system, the visible shrinkage of tumor volume in T2-FLAIR sequences was clearly associated with a reduced infiltration along the temporo-parietal portion of the sagittal stratum of Sachs. At the same time, a preferential direction towards the UF and anterior commissure fibers was easily demonstrated (Fig 6A and 6B). Standard radiological classification (insular-temporal glioma) did not highlight changes in the preferential direction of tumor progression through subcortical white matter and basal ganglia that were recorded with this system even after radio- or chemotherapy. This crucial information may help neurosurgeons and neuro-oncologists to improve individual treatment plans with the aim of restraining bilateral tumor invasion [57].

An additional advantage is the possibility to collect both quantitative and qualitative information about the kinetics of gliomas. Regarding quantitative analysis, bulky tumors showed a clear trend toward the involvement of fewer grid voxels (5.1 compared with 9 for diffuse gliomas) regardless of their volume and often within the external/cortical quadrants of the grid. This may reflect the initial stage of glioma proliferation when the centripetal infiltration process is not yet prominent. The qualitative analysis, as shown in Fig 4, is in accordance with previous reports on the preferential location of gliomas [19–23,58]. As a complement to information regarding the localization of gliomas, the real value of this system is the possibility to predict which white matter bundles are associated with the invaded voxels. For instance, the IFOF, UF, and external capsule were not the only white matter structures associated in right- and left-sided tumors harboring insular and basal ganglia grid voxels (A2-C2-S2 and A3-C2-S2), but even ATR was found as a different potential direction of tumor spread.

Based on the new information provided by the Brain-Grid classification system, we propose that this radiological-anatomical system may be a valuable tool in the field of surgical oncology, where a more precise classification and a better orientation among the deep located pathways is mandatory. Even the white matter pathways within high eloquent areas (the surgical/functional concept of the “minimal common brain”, [59]) can be analyzed with the help of the Brain-Grid classification system, including the HCP-488 template-based white matter architecture. Given the inferior plastic capacity of white matter [59], all this information could delineate an updated map of surgical resectability for each Brain-Grid voxel. A tailored surgical approach as well as the optimal timing for combined surgical and adjuvant therapies might then be based on this map of resectability and preferential direction of the white matter fiber bundles invaded by gliomas [57].

Every glioma suspected radiographically could be analyzed with the Brain-Grid classification system, integrating information about the cortical anatomy and subcortical white matter architecture easily and rapidly at any stage, pre-or postoperatively. Even in cohorts of patients retrospectively analyzed without use of individual DTI, the Brain-Grid system would be able to provide qualitative and quantitative information about the associated white matter architecture.

This universal tool may help any clinician to better understand the natural course of this disease and provide more effective tumor resection/treatment. However, this needs to be clinically validated in a larger cohort of patients (work in progress).

### Limitations

Some potential limitations need to be addressed. First, as the brain-grid classification system was defined in the MNI space, concerns may arise regarding application in the patient-specific space and the reproducibility of the system. However, the Brain-Grid classification system and subsequent analysis was reproducible using a different software in patients normalized to the MNI space. The Brain-Grid classification system should be used in the patient-specific space, an argument being the inconsistencies in metric measurement, such as millimetric infiltrations of gliomas in serial MRI, between the patient-specific space and the MNI space.

Second, the limited number of patients and the inhomogeneous population of astrocytomas and oligodendrogliomas, including both WHO grade II and WHO grade III, represent a limitation for the analysis of the results. For this reason, no clinical, histological, or functional correlations were made. However, specific clinical applications of our method with respect to symptoms and tumor-related parameters were beyond the aim of this paper, which primarily focused on a description of the classification system and its potential use in daily clinical practice. Larger cohorts of patients with homogeneous histology and the same tumor grade are necessary to show in detail differences in voxels progression/invasion. From the methodological point of view, no clear discrimination was found between tumor signal and peritumoral edema. Given that the edematous component in low grade gliomas is less prominent than in high grade gliomas (especially in bulky tumors) and that pathological cells are present even beyond the FLAIR hyperintensity [45], we considered the hyperintense signal as the tumor border at the time of radiological diagnosis. However, at the second MRI investigation, performed preoperatively after steroid administration in most cases, no differences in volume or infiltration of the pathological hyperintensity were noted, supporting the limited impact of edema in these tumors and on this classification system.

Finally, the white matter architecture included in the Brain-Grid classification system is based on a normal population. Consequently, discrimination between infiltration and displacement of fiber tracts and measurement of diffusional parameters in peritumoral white matter are not possible. Thus, the Brain-Grid classification system cannot replace patient-specific tractography performed pre-operatively.

## Conclusion

The Brain-Grid classification system provides an accurate and rapid classification of patients with gliomas that includes both cortical and subcortical anatomy. Important information about the tumor kinetics including extension and preferential direction of tumor invasion can be observed and predicted by a comparative analysis of voxels on morphological MRI and a white matter architecture atlas like that included in the Brain-Grid classification system. This new integrated classification of gliomas can potentially help clinicians to plan tailored tumor resection and target volume for radiotherapy based on a prediction of white matter invasion.

## Supporting information

S1 Appendix Brain-Grid-DTT atlas 1. Description of the Data: This file includes general instructions for the reader to use the Brain-Grid classification system and the complementary tractographic atlas generated from the HCP dataset. The atlas provided 3-D anatomical and functional information of the major commissural and projection fiber systems.
(PDF)

S2 Appendix Brain-Grid-DTT atlas 2. This file includes the second part of the complementary tractographic atlas generated from the HCP dataset. The atlas provided 3-D anatomical and functional information of the major associative fiber systems of the brain.
(PDF)

## Abbreviations

AC: Anterior commissure
AF: Arcuate fasciculus
Ci: Cingulum
CC: Corpus callosum
DSI: Diffusion spectrum imaging
DTT: Diffusion tensor tractography
FAT: Frontal aslant tract
FM: Forceps major
Fo: Fornix
ILF: Inferior longitudinal fasciculus
IFOF: Inferior fronto-occipital fasciculus
MLF: Middle longitudinal fasciculus
OR: Optic radiation
ROI: Region of interest
SSS: Sagittal stratum of Sachs
SLF: Superior longitudinal fasciculus
TR: Thalamic radiation
UF: Uncinate fasciculus
VOF: Vertical occipital fasciculus
Native space: patient-specific space
Montreal Neurological Institute (MNI) space: space defined by the MNI template
Spatial normalization: the process to transform images from native to the MNI space
