# Supplementary material for: A novel radiological classification system for cerebral gliomas: The Brain-Grid
Source: PLoS One. 2019 Jan 24;14(1):e0211243. doi: 10.1371/journal.pone.0211243 (PMC6345500; doi:10.1371/journal.pone.0211243)
Supplement: S1 Appendix — Description of the Data: This file includes general instructions for the reader to use the Brain-Grid classification system and the complementary tractographic atlas generated from the HCP dataset. The atlas provided 3-D anatomical and functional information of the major commissural and projection fiber systems. (PDF) [file pone.0211243.s001.pdf]

How to read the Brain Grid white matter atlas.

The aim of this atlas is to provide an observational tool for a white matter classification based of intracerebral gliomas. The figures provided below show the position of every major white matter fiber system within the Brain Grid. For each bundle or system six different views are provided to help the reader with 3-D orientation. The MNI space coordinates on each perspective reproduce the main distribution of each bundle within the average space as already described by other authors [1,2,3]. Labels with the numbers and letters for each brain grid unit are displayed together with the MNI coordinates. The description of each white matter bundle or system includes the type of the system according to the general organization of cerebral white matter [4], the anatomical description of the main course of the pathway with a more detailed attention to the cortical terminations or origins, the distribution within the BG system with labels of the terminations and the stem of each white matter bundle. The described functions of each system are also provided according to an extensive review of the literature.

The Brain grid classification provided here is labelled according to the macrovoxels involved (1 letter and 1 number) rather than microvoxels (3 letters and 3 numbers). We believed that at this stage the major advantage of this tool would be the visual orientation within the grid, better than a systematic labelling of each quadrants as described in the manuscript. The microvoxels can however be extrapolate from the images for a more specific distribution of the fiber systems stems and terminations.

The reader should be aware that the terms “terminations or origin” are used in a speculative way for two reasons: first the real cortical terminations cannot be displayed with the actual level of resolution provided by diffusion tractography. Second, the fiber systems displayed rather work in a bidirectional parallel way and the terms origin and terminations lose strength accordingly. However, since the major atlases (tractographic and white matter dissection) used as references [1-3,5-9] provided also a better and more detailed description of the distal portion of the superficial white matter organization, it seems correct to push the topographical analysis to the closest subcortical regions. This hopefully may add some additional information to the surgical/ application of this new classification tool.

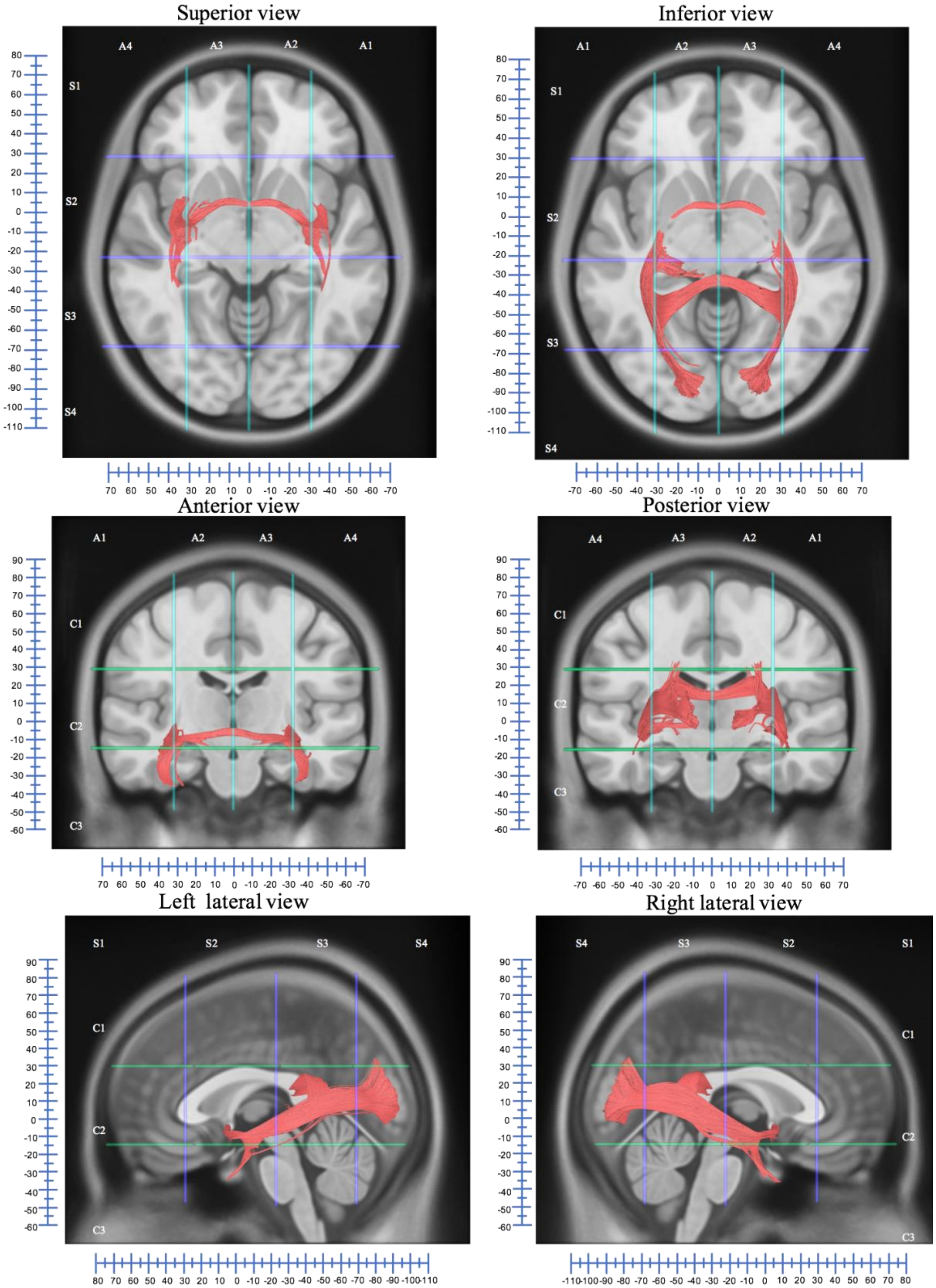

Figure 7. Anterior commissure. [1,2,5,6,8-11]

Type: Commissural fiber system.

Anatomy: The anterior commissure connects the anterior, ventral temporal lobes (including the amygdala) and temporo-occipital junctions of the two hemispheres. The body (stem) of the anterior commissure crosses the midline in the anterior wall of the third ventricle and bifurcates more laterally into anterior and posterior branches on both sides. The anterior

branch passes through the subinsular regions to reach the temporal lobe, where it gives extensions to the temporal pole (anteriorly) and medial part of the occipital lobe (posteriorly).

Brain Grid: Stem: A2-A3, C2, S2; Bifurcation: A1-A4, C2, S2; Temporal anterior: A1-A4, C3, S2; Temporo-Occipital: A2-A3, C2, S4

Functions: AC integrates the interhemispheric transfer of visual, auditory, olfactory, and gustative information between temporal lobes.

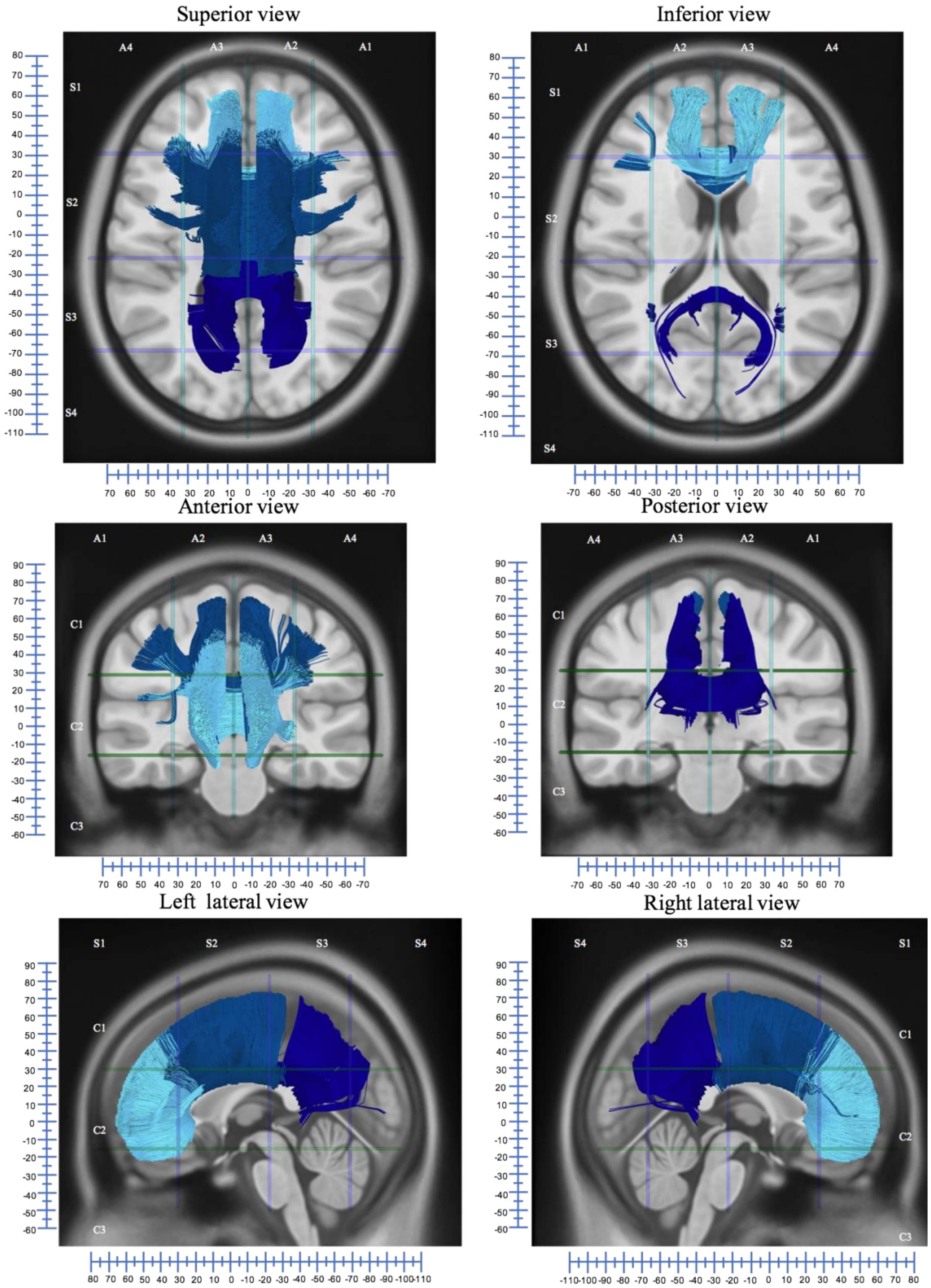

Figure 8. Corpus Callosum (CC). [1,5,8,9,13-17]

Type: Commissural fiber system.

Anatomy: The genu of the CC mediates connectivity between the prefrontal regions (turquoise), the body (blue) connects the premotor and supplementary motor area (SMA) regions, and posteriorly connects the primary sensorimotor areas. The splenium (dark blue) contains fibers originating from the parietal, temporal, and occipital association cortices.

Brain Grid: Genu from A2-A3, C2, S2 to A2-A3, C1-C2-C3, S1. Body from A2-A3, C2, S1-S2-S3 to A2-A3, C1, S1-S2-S3. Splenium from A2-A3, C2, S3 toA2-A3, C1-C2, S4.

Functions: The CC integrates the right and left hemispheric neocortical networks. The CC is involved with such varied processes as bimanual sensorimotor coordination, auditory and visuo-motor coordination, and Injury to the callosal fibers can result in a variety of disconnection syndromes, all of which are characterized by some aspect of impaired sensory or cognitive integration.

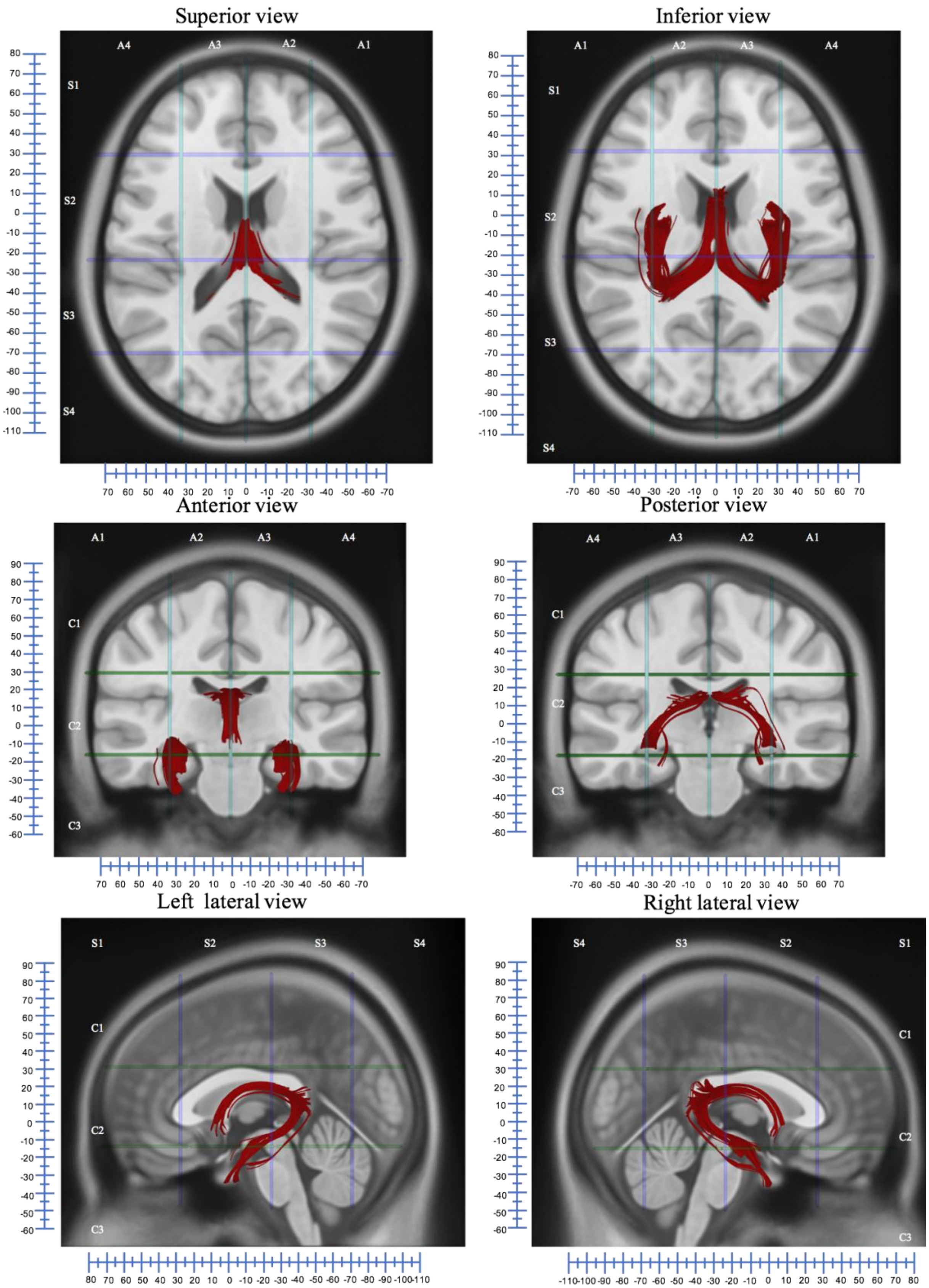

Figure 9. Fornix. [1,3,5,7-9,18,19]

Type: Commissural fiber system.

Anatomy: The fornix connects the medial temporal lobe to the mammillary bodies and hypothalamus, mammillary bodies to anterior nuclei of the thalamus and hippocampus to the septal nuclei and nucleus accumbens.

Brain Grid: From A2-A3, C2, S2 to A1-A2-A3-A4, C2-C3, S2.

Functions: The fornix integrates complex cognitive information within the limbic system of both hemispheres and is involved in memory functions, both formation and recall.

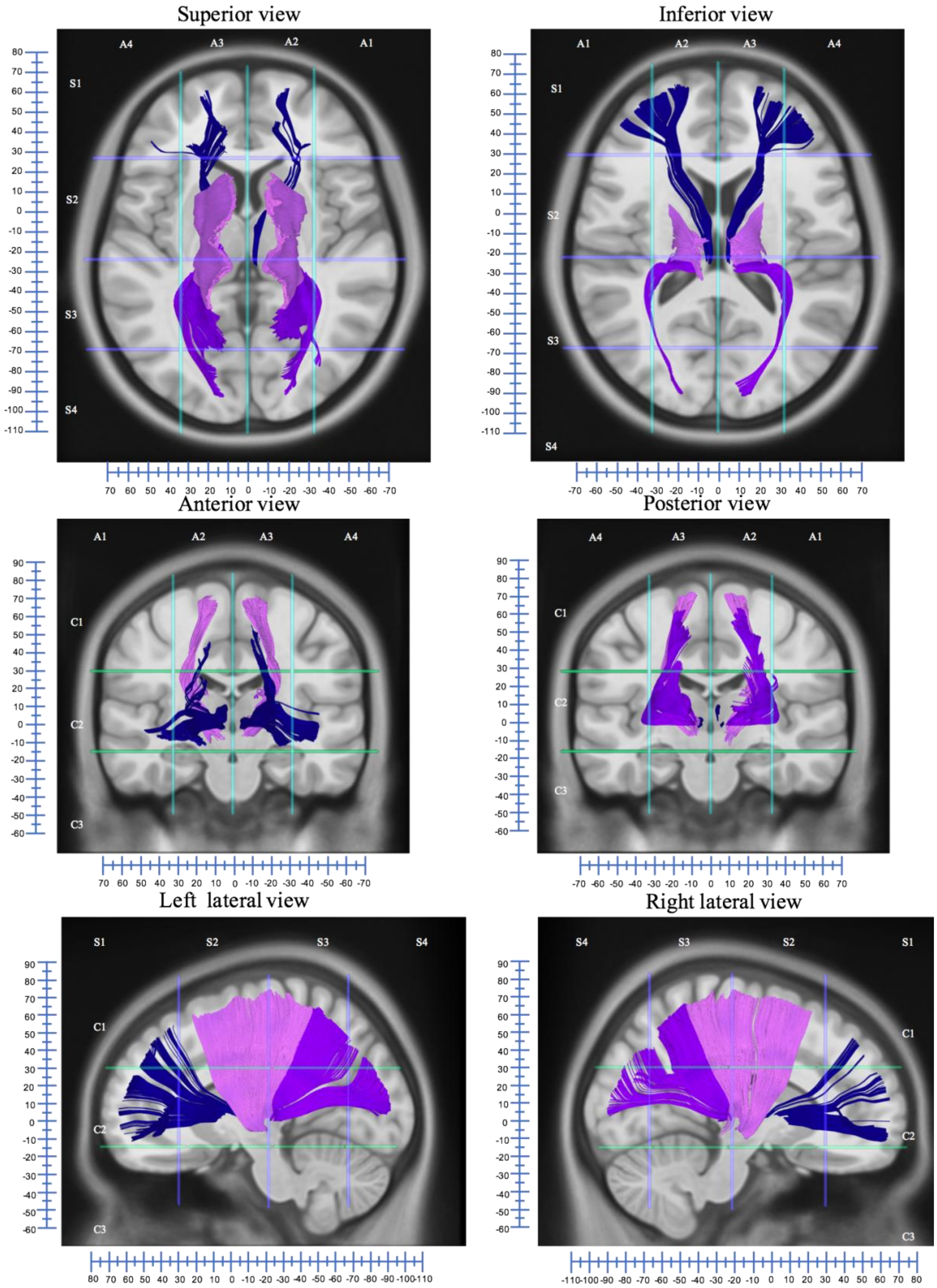

Figure 10. Thalamic radiation. [1,3,5,6,8,9,20-22]

Type: Projection fiber system.

Anatomy: The thalamic radiation connects the thalamus and the cortex of the fronto-parietal and occipital lobe. These fibers converge into the internal capsule, located between the lentiform nucleus (putamen and globus pallidus) and the thalamus– caudate nucleus regions. The anterior part of the thalamic radiation (dark blue) connects the anterior nuclear group of the thalamus and the midline nuclear group of the thalamus with the frontal lobe through the anterior thalamic peduncle, the anterior limb of the internal capsule and other parts of the cerebral white matter among other types of sensory connections between the thalamus and cortex. The superior thalamic radiation (pink) includes the upper part of the corona radiata (fronto-parietal) merged with the fibers of the cortico-spinal tract. The posterior thalamic radiation (lavender color) includes a portion of the optic radiation. The thalamic fibers all penetrate the internal capsule, where the cortico-spinal connection occupies the more lateral regions.

Brain Grid: Anterior thalamic radiation from A2-A3, C2, S2 to A1-A2-A3-A4, C1-C2, S1; Superior thalamic Radiation from A2-A3, C2, S2-S3 to A2-A3, C1, S2-S3; Posterior thalamic radiation from A2-A3, C2, S2 to A2-A3, C1-C2, S3-S4.

Functions: The thalamic radiation integrates the complex sensory-motor information for high order abilities, are among others vision and auditory related functions, attention, wakefulness, and arousal. Specifically, the ATR damage has been associated with multiple psychiatric and psychological syndromes such as schizophrenia and bipolar disorders.

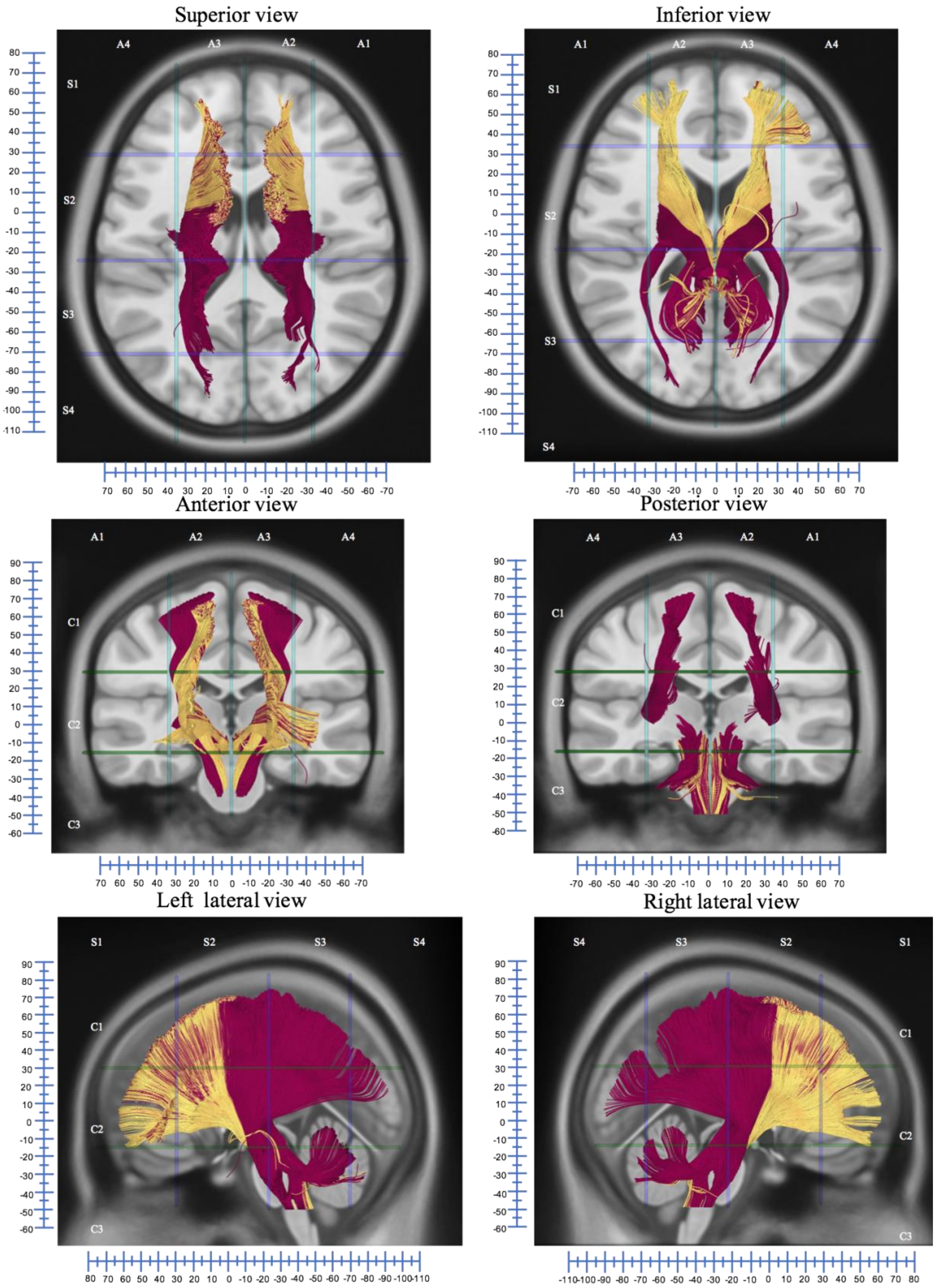

Figure 11. Internal Capsule and Corona radiata. [1,3,6,23,24]

Type: Projection fiber system.

Anatomy: The internal capsule and corona radiata connect the thalamus to the cerebral cortex (ascending fibers form) and the fronto-parietal cortex to subcortical nuclei and spinal cord (descending fibers). Fibers that run from the internal

capsule (central core region) to the cortex form a “fan-shaped” structure (corona radiata). The internal capsule is bordered by the thalamus and caudate medially and by the lenticular nucleus laterally. The internal capsule is here divided into anterior and posterior limbs that are joined at the genu. The anterior part (gold) includes the anterior limb of the capsule and the anterior half of the genu. It carries fronto-pontine and thalamo-frontal (anterior thalamic peduncle) fibers and from the prefrontal and precentral region. The posterior part (maroon color) of the internal capsule is formed by fibers from the precentral and postcentral region, and has a slightly oblique postero-anterior direction. The retrolenticular portion of the internal capsule is composed of fibers coming from the posterior parietal and occipital cortex, and is oriented in a sagittal plane in their passage toward the internal capsule.

Brain Grid: Anterior portion from A2-A3, C2, S2 cranially to A1-A2-A3-A4, C2, S1 and A2-A3, C1, S1-S2; caudally to A2-A3, C3, S3-S4. The posterior portion from A2-A3, C2, S2-S3 cranially to A2-A3, C1-C2, S2-S3-S4, caudally to A2-A3, C2-C3, S2-S3-S4.

Functions: It integrates information involved in perceptual and motor functions and other higher cognitive functions.

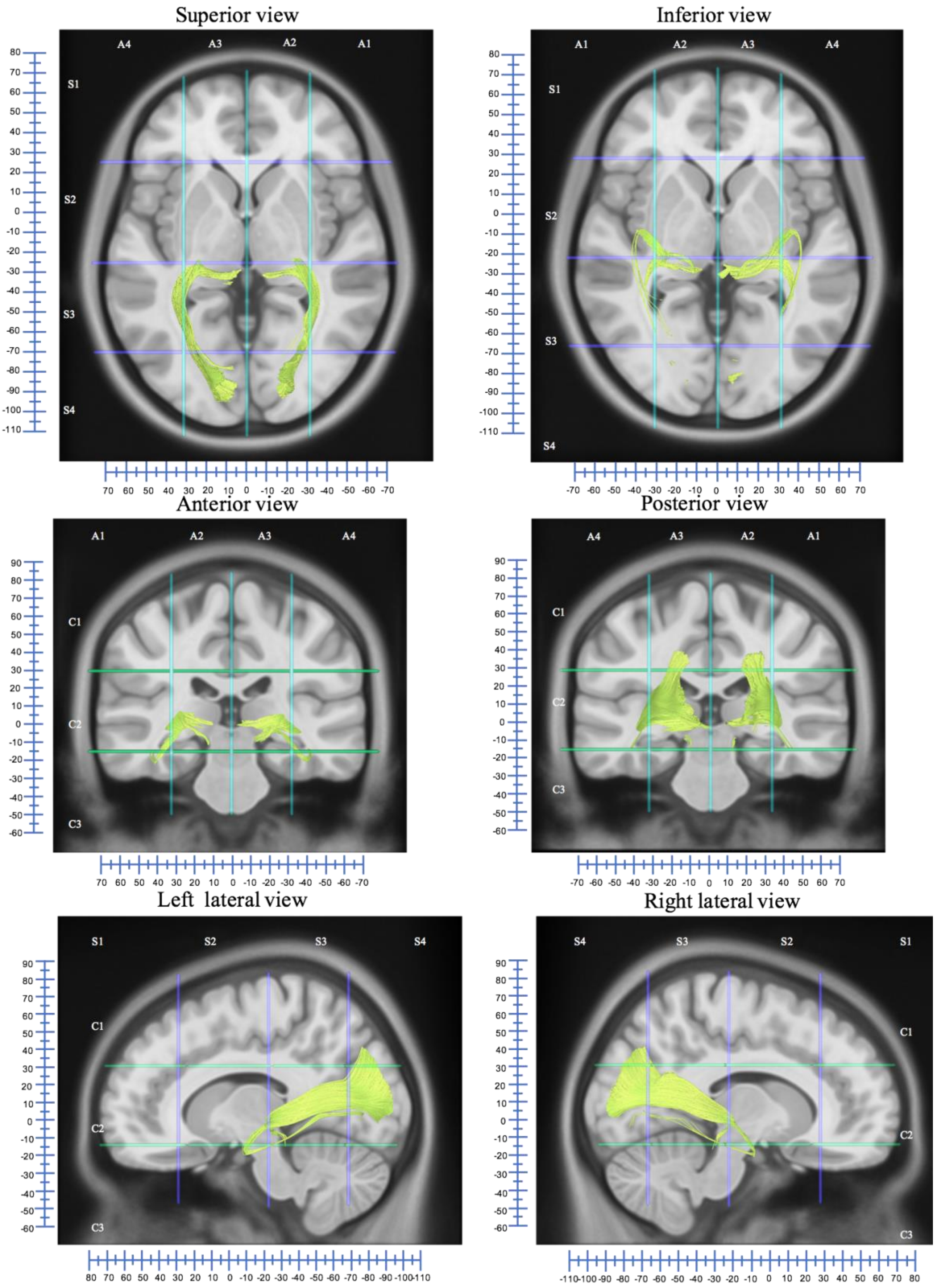

Figure 12. Optic radiation. [3,6-9,25]

Type: Projection fiber system.

Anatomy: The OR connects the lateral geniculate body of the thalamus and the medial occipital areas within or close to the calcarine fissure. The main portion of these bundles origins from the postero-lateral portion of the thalamus and follows the course of the posterior thalamic radiation, through the lateral wall of lateral ventricle to the medial portion of

the occipital lobe (calcarine fissure, V1, V2, V3). The most anterior/ventral portion of the OR (Meyer's loop) enters the temporal stem, passes over the roof of the temporal horn, and proceeds posteriorly along the lateral margin of the temporal horn.

Brain Grid: Main stem of OR from A2-3, C2, S3 to A2-A3, C1-C2, S4. The Meyer´s loop A2-A3, C2, S2 through A1-A4, C3, S2-S3, to A2-A3, C2, S4.

Functions: The OR integrates visual information (which originate from the retina of the eye transmitted to the thalamus through the optic tract) and the visual cortex. Lesion of this tract would create different patterns of visual field deficits.

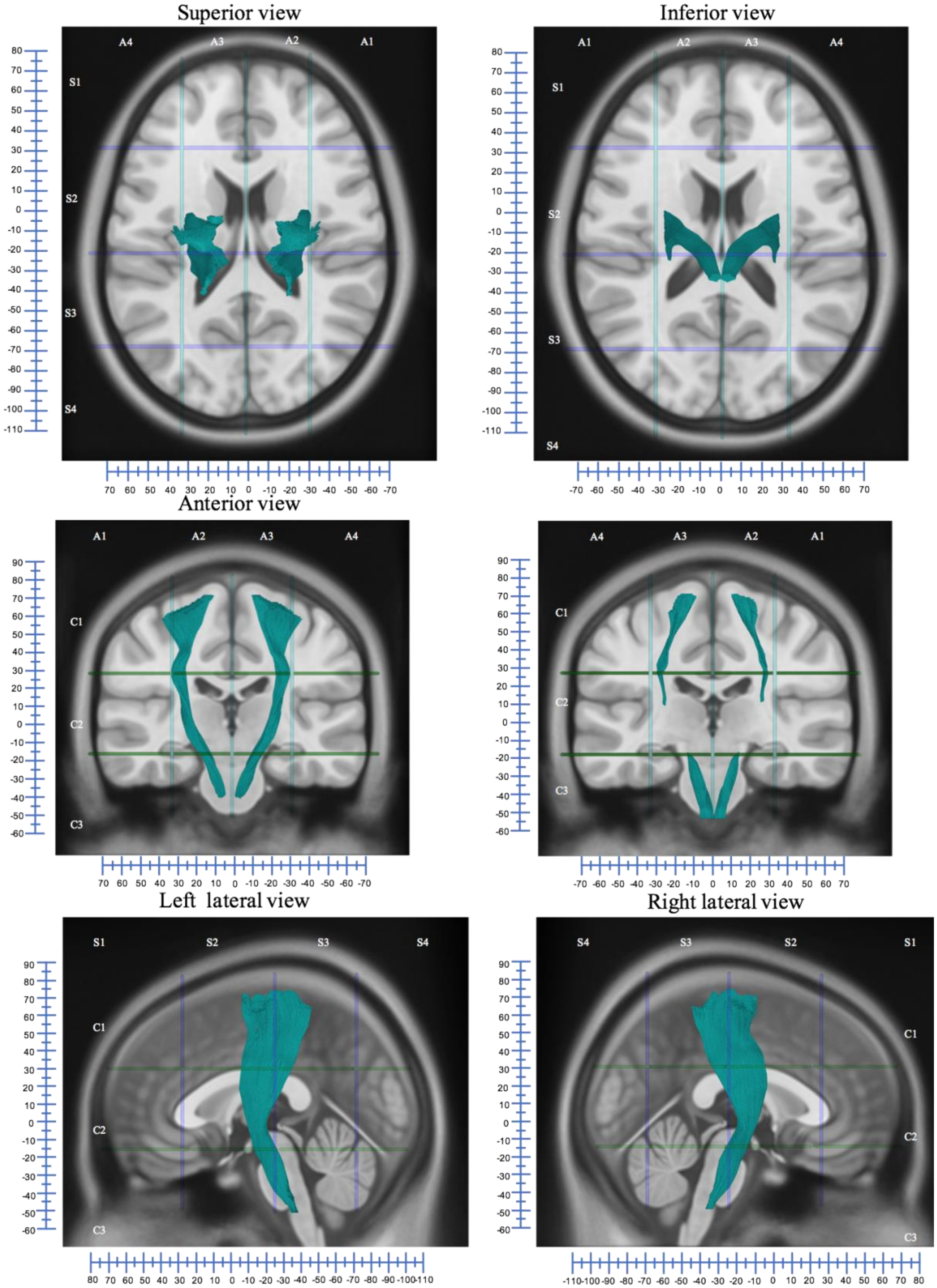

Figure 13. The Pyramidal tract. [1,3-5,7-9,24,26]

Type: Projection fiber system.

Anatomy: The pyramidal tract connects Cerebral cortex close to the central sulcus and the brainstem/ spinal cord. It contains upper motor neurons that originate in the cerebral cortex and terminate in the spinal cord (corticospinal) or brainstem (corticobulbar). Nerves emerge in the cerebral cortex, pass down and the majority of the fibers cross the midline

in the medulla oblungata, and travel as part of the spinal cord until they synapse with lower motor neurons and interneurons in the grey column of the spinal cord.

Brain Grid: From A2-A3, C1, S2-S3 to A2-A3, C3, S2-S3.

Functions: The pyramidal tract integrates sensory and motor information and represents the highest order of motor function in human, mostly involved in control of fine, digital movements.

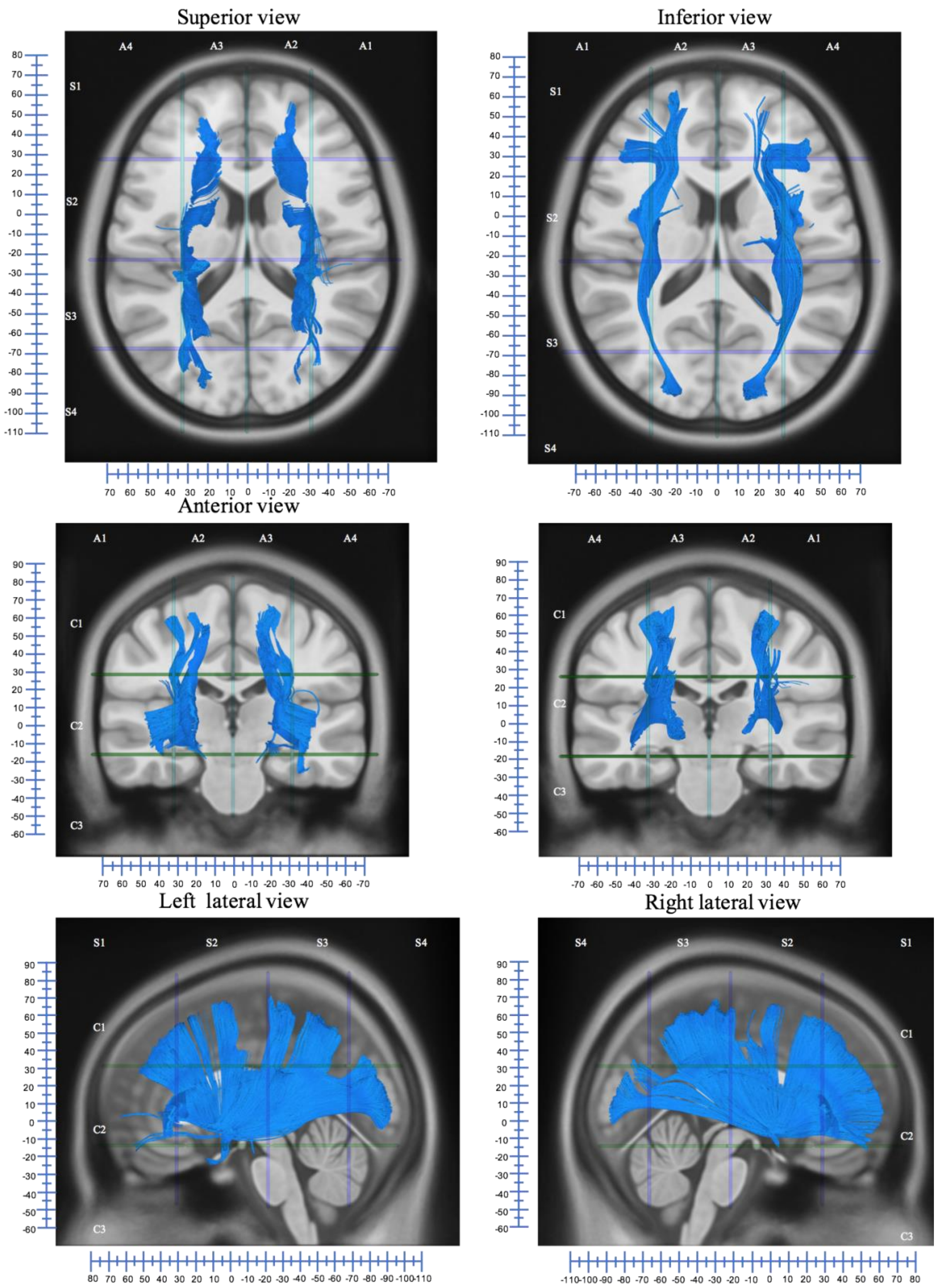

Figure 14. External Capsule. [3,4,7-9]

Type: Cortico-Striatal fiber system (dorsal), Associative fiber system (ventral)

Anatomy: The external capsule is classically described as a layer of fibers situated between the putamen medially and the claustrum laterally. Its fibers convey in a "fan" like shape from the ventral and medial pre-frontal cortex, ventral

premotor cortex, pre- central gyrus, the rostral superior temporal region, and the infero-temporal and preoccipital regions to the central core of the brain. The dorsal portion of the external capsule is considered a cortico-striatal fiber system which includes fibers of the corona radiata that direct centripetally to the dorsal claustrum and putamen where these fibers end. The ventral portion of the external capsule (is considered an associative fiber system) contains fibers from the uncinate fasciculus and longitudinal fibers of the inferior occipito-frontal fasciculus (see below).

Brain Grid: Dorsal terminations A2-A3, C1, S2-S3; ventral anterior terminations A1-A2-A3-A4, C1-C2, C3, S1-S2; ventral posterior terminations: A3-A4, C1-C2, S3-S4.

Functions: The External capsule with its cortico-striatal/associative pathways provide an important link that enable different regions with the basal ganglia to contribute to motor control, cognition, and emotion.
