# Supplementary material for: A novel radiological classification system for cerebral gliomas: The Brain-Grid
Source: PLoS One. 2019 Jan 24;14(1):e0211243. doi: 10.1371/journal.pone.0211243 (PMC6345500; doi:10.1371/journal.pone.0211243)
Supplement: S2 Appendix — This file includes the second part of the complementary tractographic atlas generated from the HCP dataset. The atlas provided 3-D anatomical and functional information of the major associative fiber systems of the brain. (PDF) [file pone.0211243.s002.pdf]

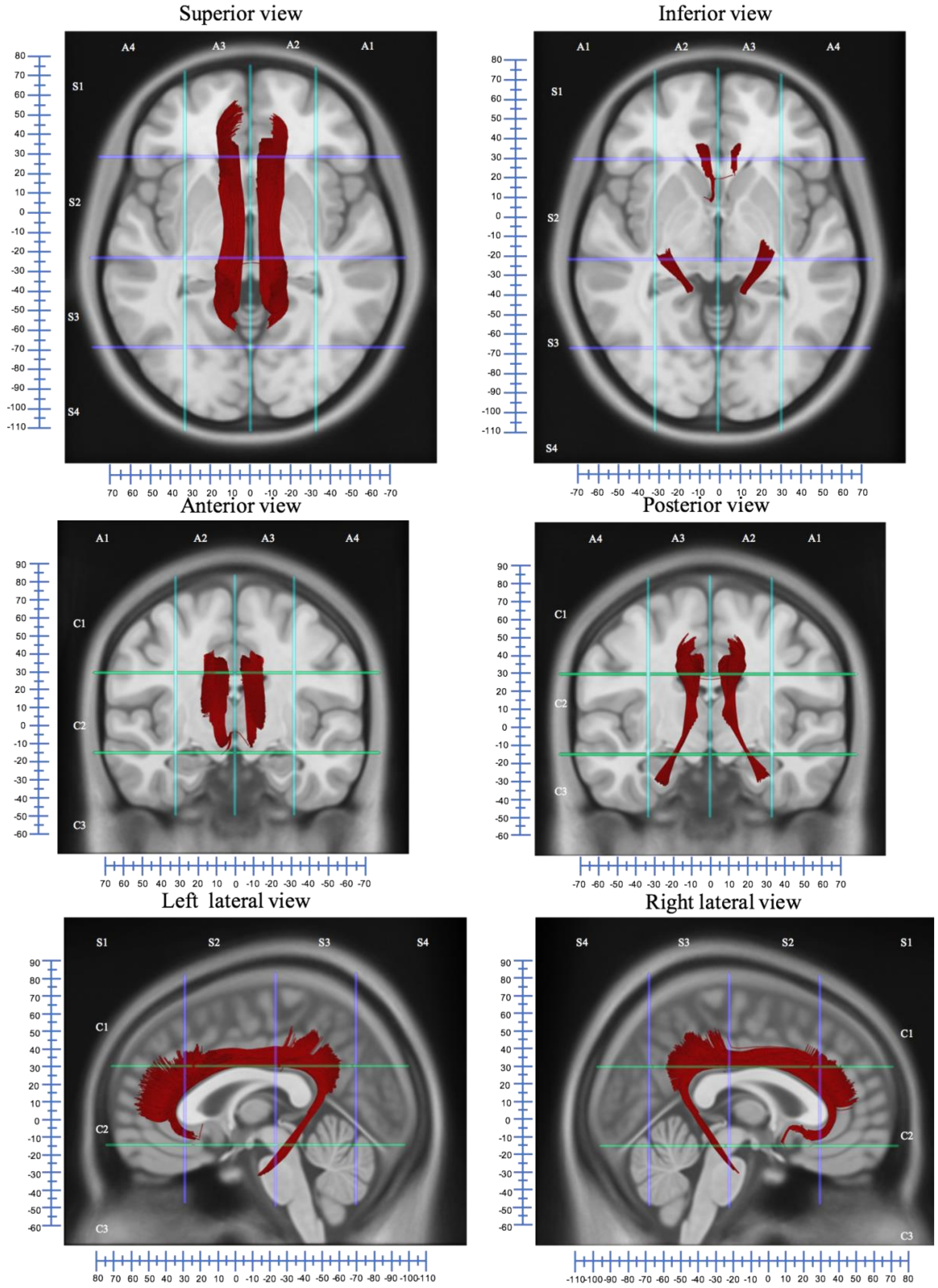

Figure 15. Cingulum. [1,5-9,27]

Type: Associative fiber system.

Anatomy: The cingulum runs within the cingulated gyrus all around the corpus callosum. It contains fibers of different length, the longest of which runs from the anterior temporal gyrus to the orbitofrontal cortex. The short U-shaped fibers connect the medial frontal, parietal, occipital, and temporal lobes and different portions of the cingulated cortex.

Brain Grid: A2-A3, C2, S1-S2 to A2-A3, C3, S2-S3

Functions: The cingulum integrates high order information within the limbic system and is involved in attention, executive functions, decision-making, memory and emotion processing.

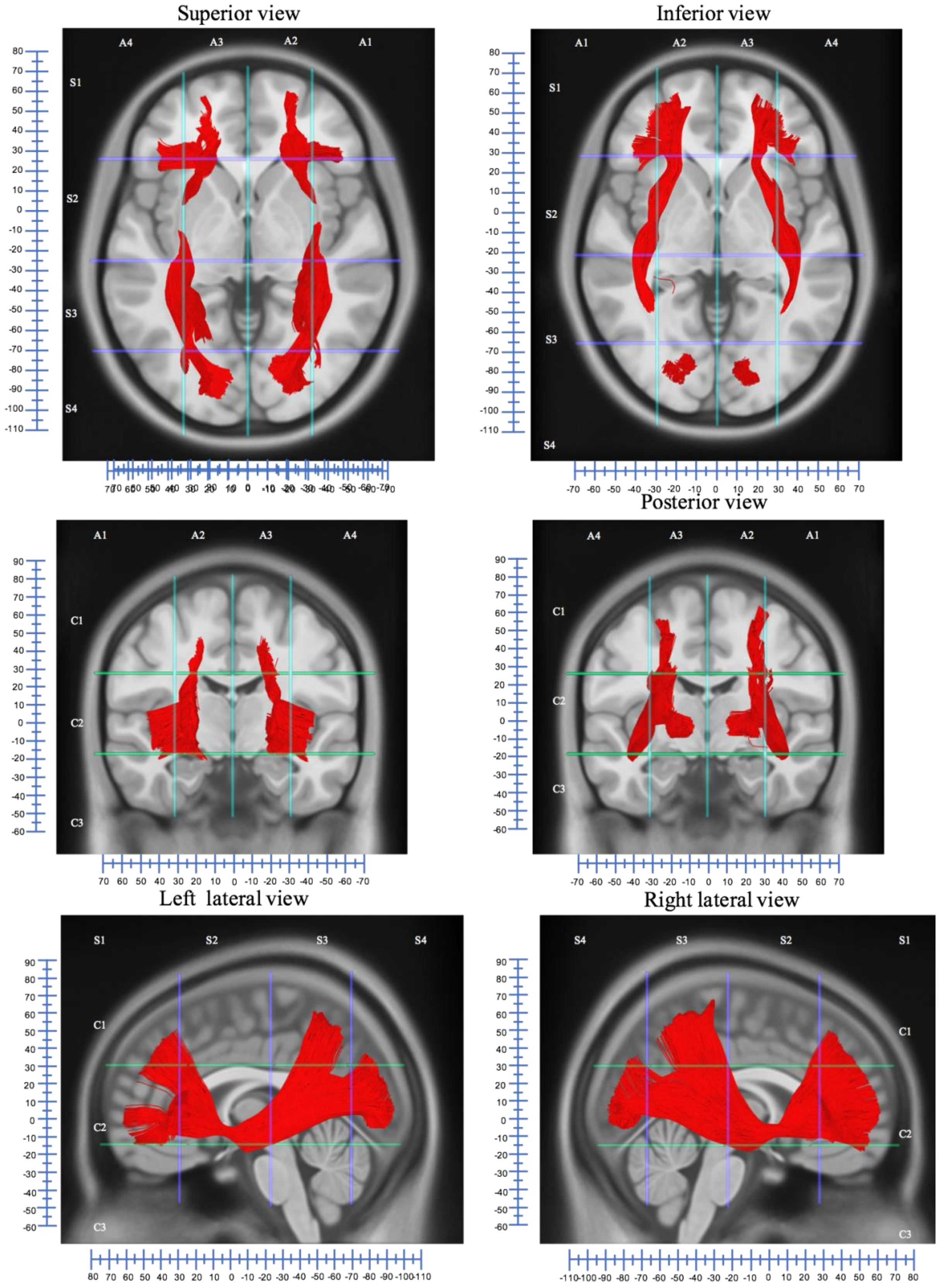

Figure 16. Inferior Fronto-Occipital Fasciculus (IFOF). [1,5,6,8,27-35]

Type: Associative fiber system

Anatomy: IFOF connects the ventral and dorsal occipital lobe, the parietal lobe and the orbitofrontal cortex. In its longitudinal course the inferior fronto-occipital fasciculus runs within the occipital lobe lateral and parallel to optic radiation within the Sagittal Stratum of Sachs. On approaching the anterior temporal lobe, the fibers of the IFOF gather

together and enter the external capsule dorsally to the fibers of the uncinate fasciculus. It has terminations within the anterior portion of the superior frontal gyrus, frontal pole and orbito-frontal cortex more laterally.

Brain Grid: Anterior terminations from A1-A2-A3-A4, C1-C2, S1-S2 to the posterior terminations A2-A3-C1-C2, S3-S4.

Functions: IFOF integrates human language functions, particularly lexico-semantic processing but also non-language related tasks. It participates to reading, attention and visual processing.

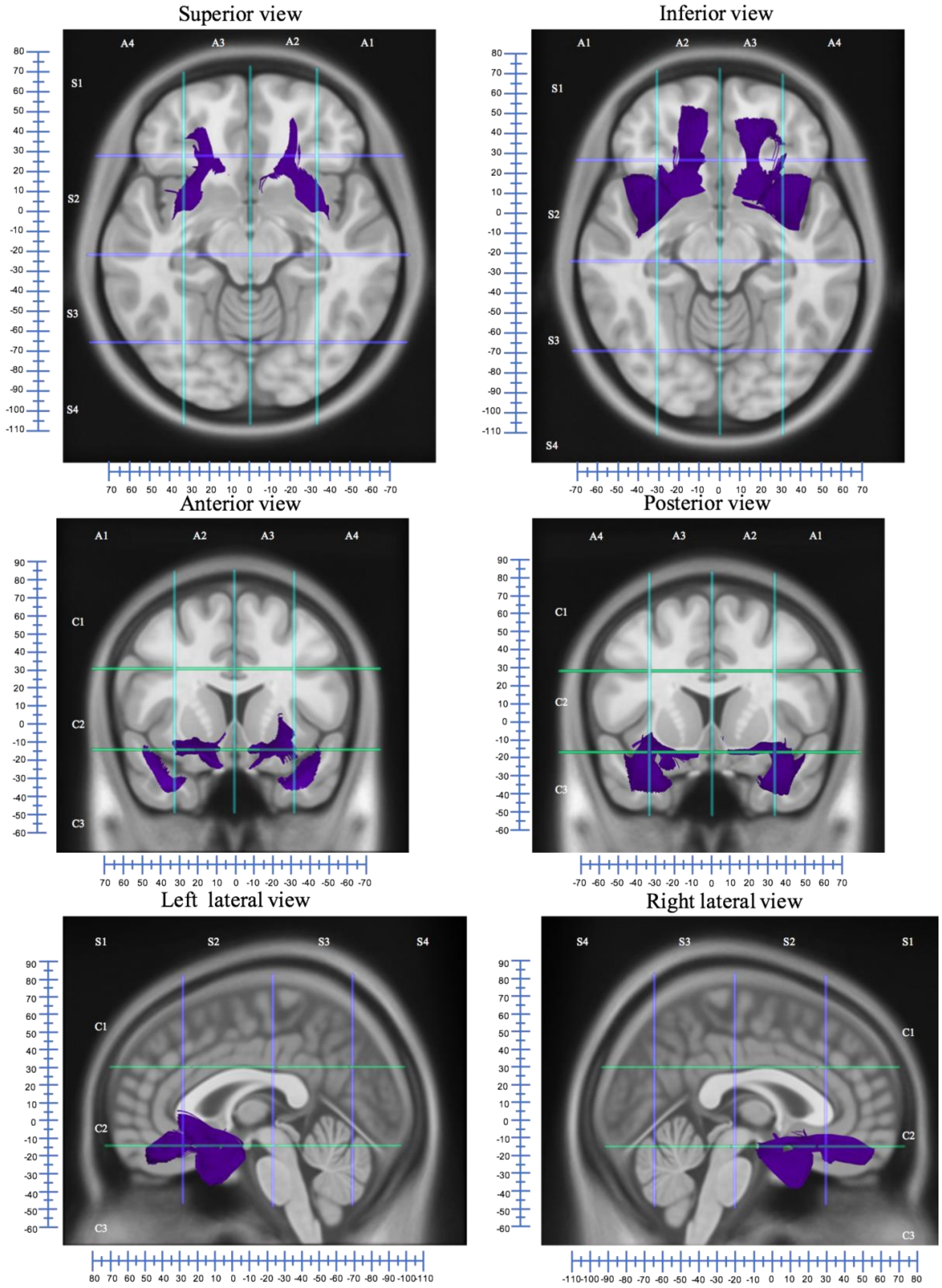

Figure 17. Uncinate fasciculus (UF). [1,3,5,6,34,36,37]

Type: Associative fiber system

Anatomy: UF connects the anterior temporal lobe with the medial and lateral orbitofrontal cortex. The UF originates from ventral frontal areas, ventral and lateral to IFOF origins. It remains ventrolateral to the IFOF as it passes into the temporal stem region, and hooks away inferiorly and then anteriorly to its terminations within the anterior temporal regions.

Brain Grid: Frontal terminations: A2-A3, C2-C3, S1-S2 to temporal terminations A1-A2-A3-A4, C3, S2.

Functions: The UF integrates high order information with a pivotal role in episodic memory, language and social emotional processing.

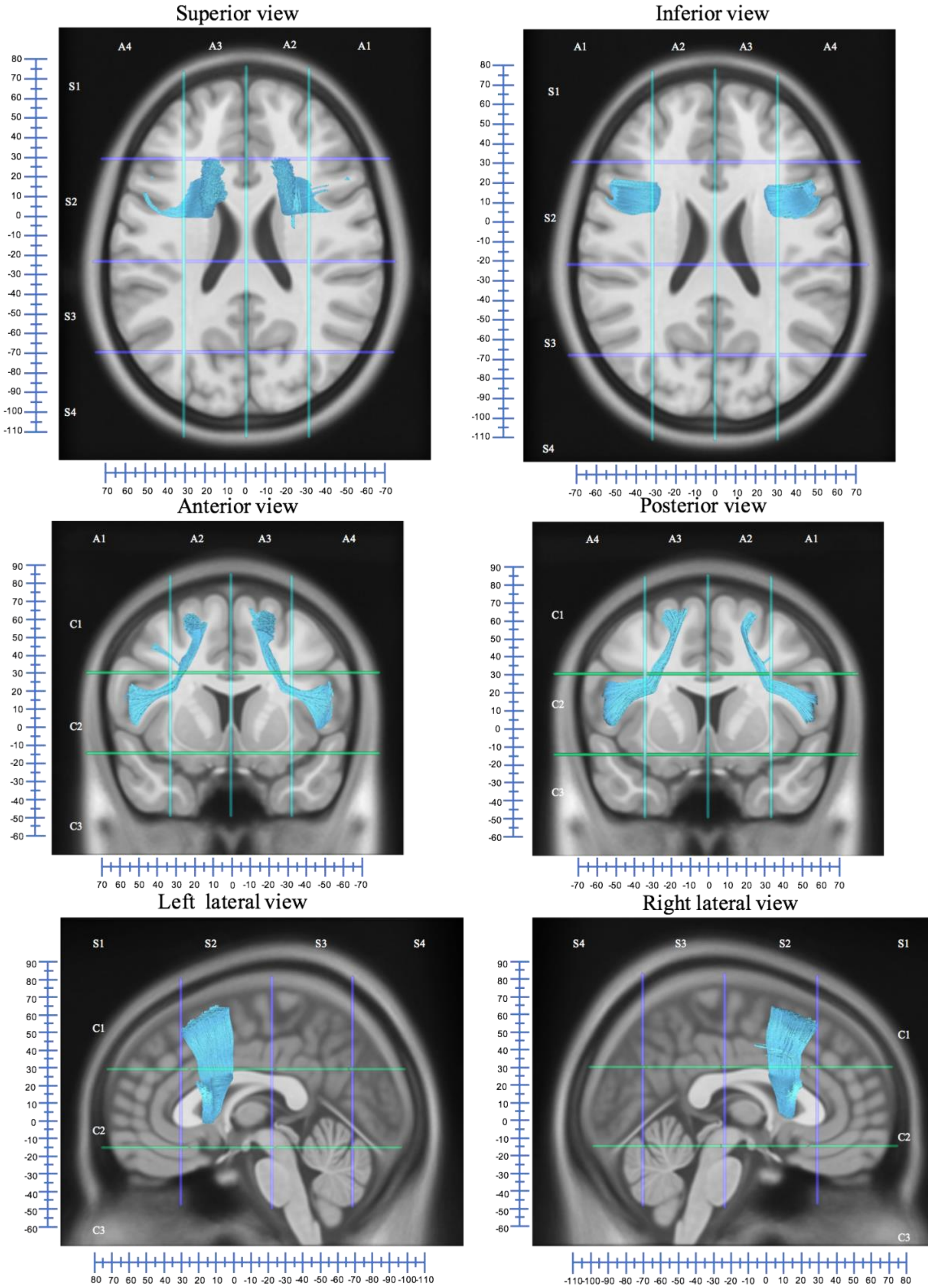

Figure 18. Frontal Aslant Tract (FAT). [1,6,33,38-41]

Type: Associative fiber system

Anatomy: The FAT connects with an oblique fiber pathway the most posterior part of Broca's territory (i.e., pre-central cortex and pars opercularis) in the inferior frontal gyrus to the supplementary motor area (SMA) and pre-SMA in the superior frontal gyrus

Brain Grid: Superior terminations: A2-A3, C1, S2; inferior terminations: A1-A4, C2, S2.

Functions: The frontal aslant tract integrates information involved in motor planning, including vocalization and speech. The FAT has also been critically involved in the network sub-serving constructional praxis in patients with Alzheimer Disease.

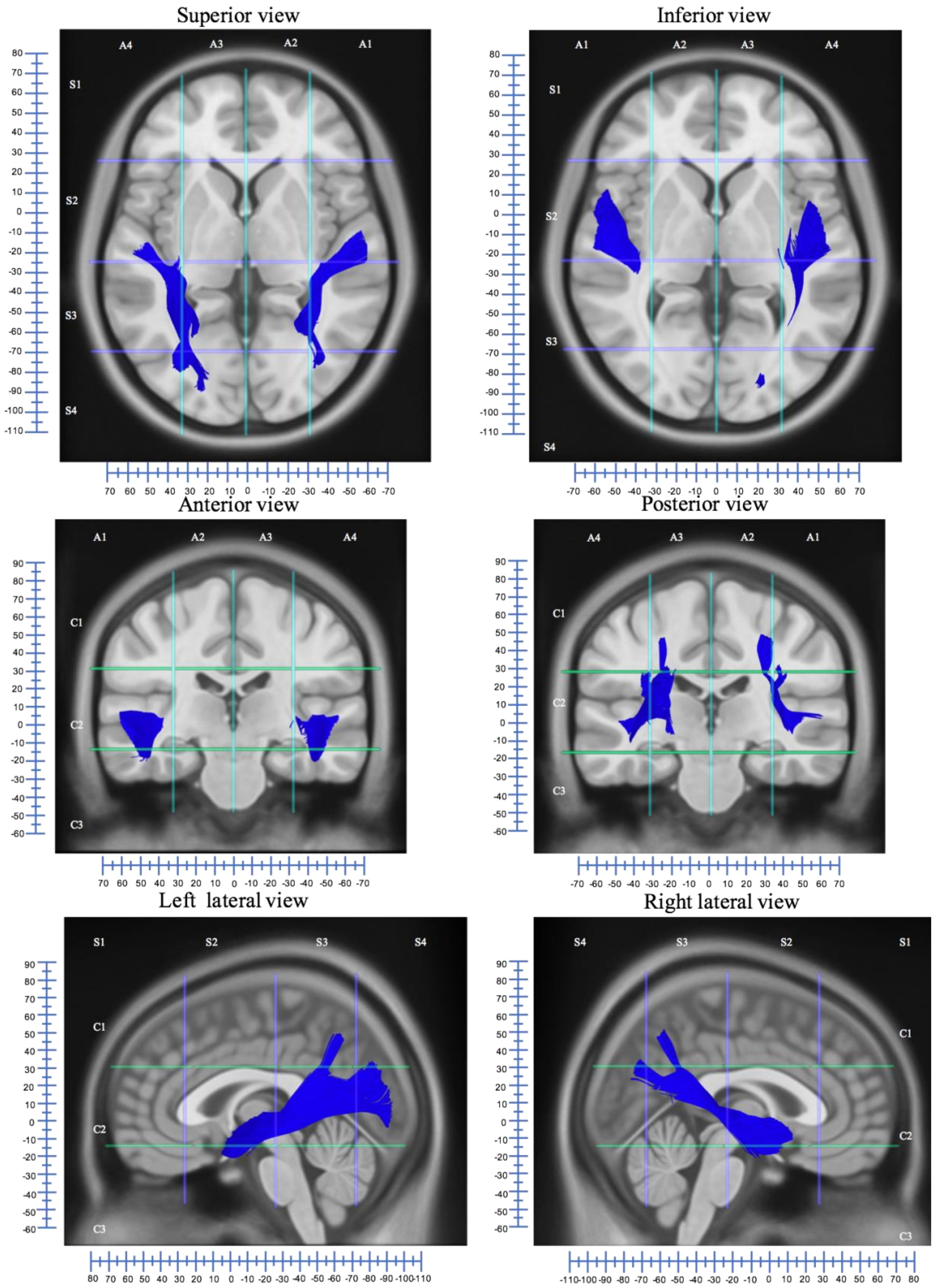

Figure 19. Middle Longitudinal Fasciculus (MLF). [9,42-49]

Type: Associative fiber system

Anatomy: The MLF connects the superior temporal gyrus and temporal pole with the supramarginal gyrus, the superior parietal lobule and the precuneus in the parietal lobe, and the cortices of the cuneus and the ventral occipital region in the

occipital lobe. From the superior temporal gyrus, it runs posterior and medial, lateral in respect to the IFOF within the Sagittal Stratum of Sachs.

Brain Grid: Anterior terminations from A1-A4, C2, S2 to posterior terminations A1-A2-A3-A4, C1-C2, S3-S4

Functions: The MLF is believed to integrate language, attention and integrative higher level visual and auditory processing associated functions.

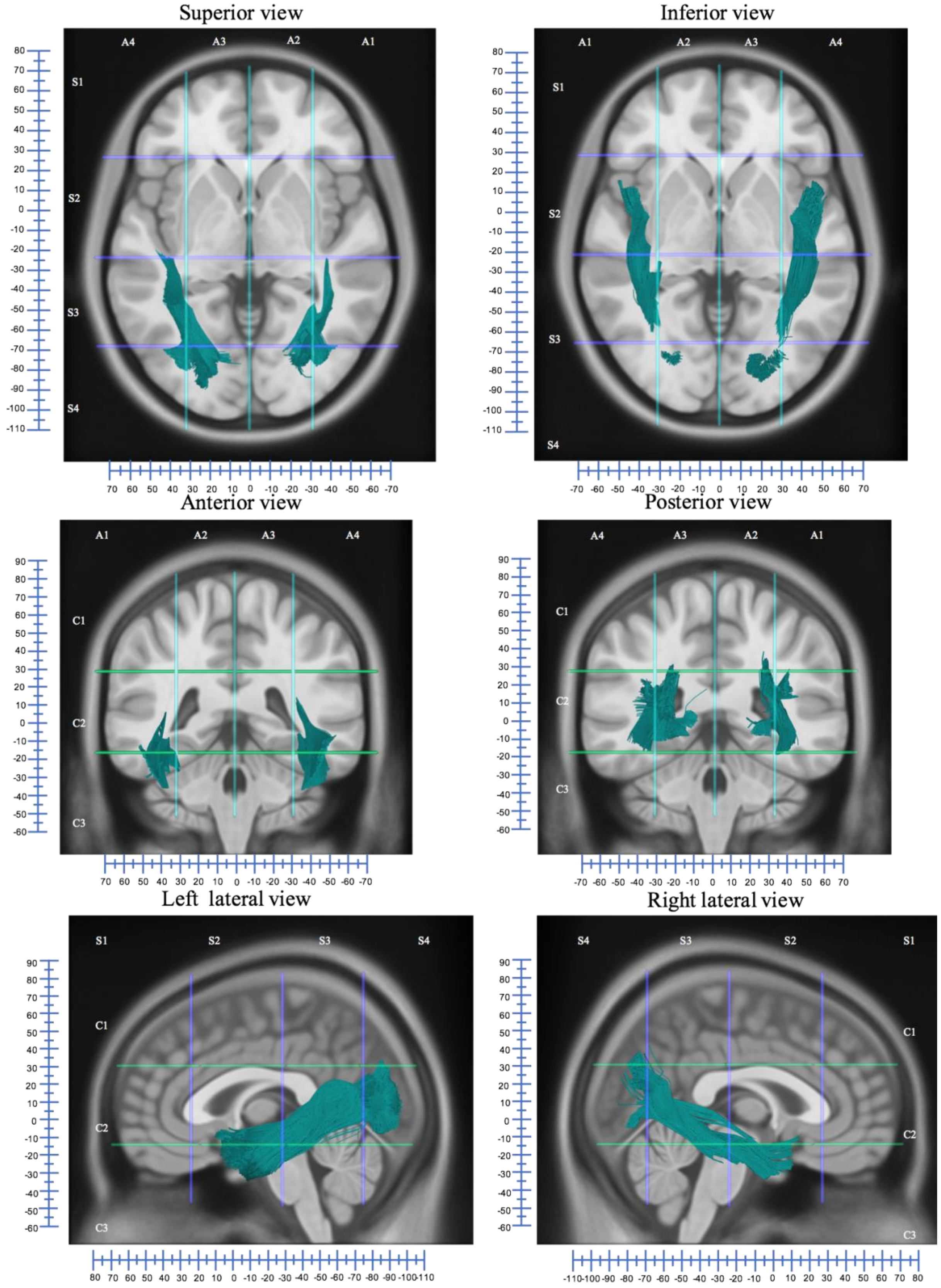

Figure 20. Inferior Longitudinal Fasciculus (ILF). [1,6,8,30,31,40,50,51-55]

Type: Associative fiber system

Anatomy: The ILF connects with long and short fibers the occipital and temporal lobes. The long fibers are medial to the short fibers and connect extrastriate visual areas to the amygdala and hippocampus medially, to the temporal pole anterior

and to the superior, medial and inferior temporal gyri laterally. It runs longitudinal and ventral within the Sagittal Stratum of Sachs, lateral and caudal to the MLF.

Brain Grid: anterior temporal terminations from A1-A4, C2-C3, S2 to the occipital terminations A1-A2-A3-A4, C1-C2, S3-S4.

Functions: The ILF integrates information from highly specialized modular visual areas with activity in anterior temporal territory important for memory and emotions. It is involved in face recognition visual perception, reading, visual memory and other functions related to language.

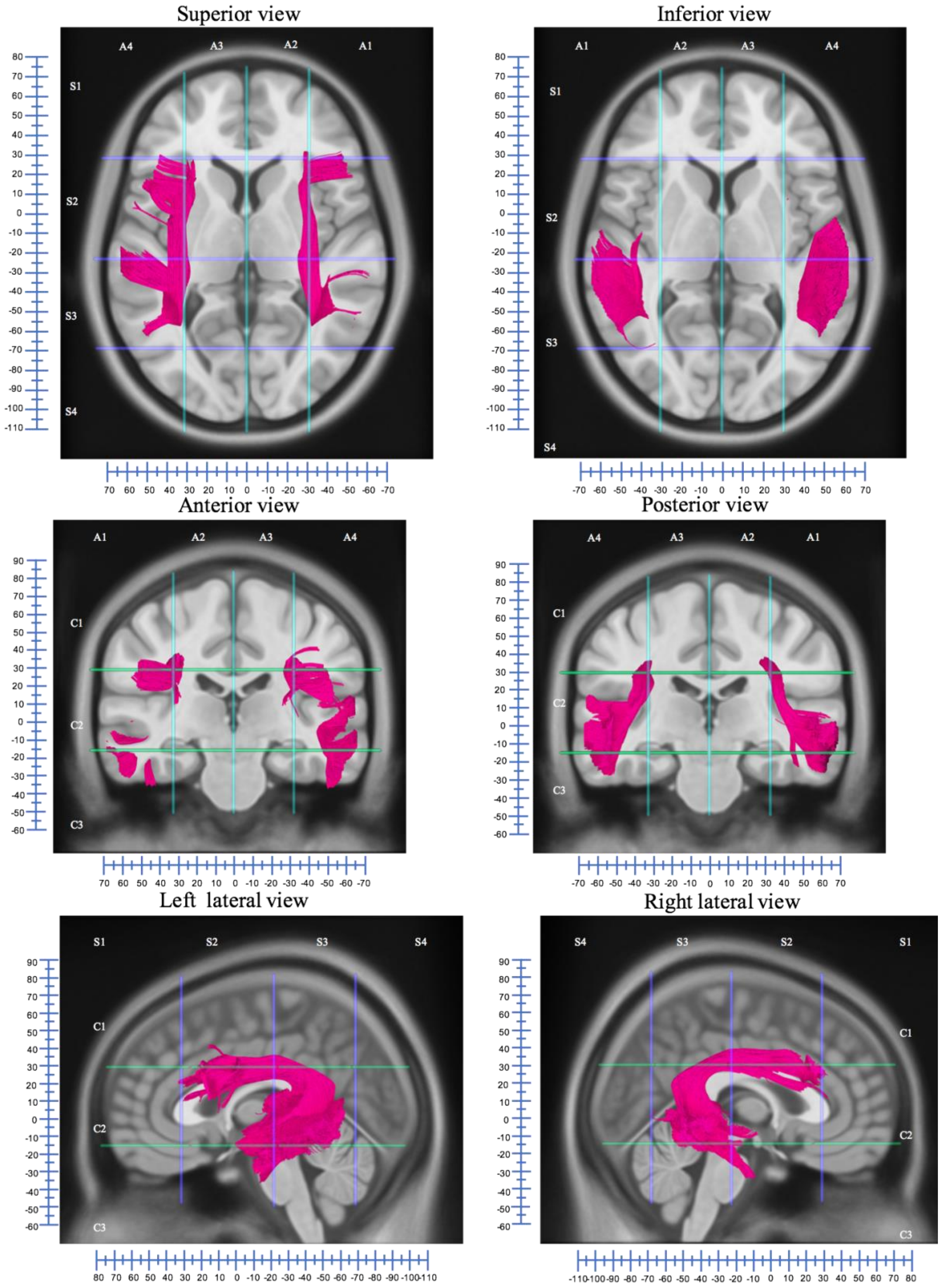

Figure 21. Arcuate Fasciculus (AF) or Arcuate Segment of the Superior Longitudinal Fasciculus (SLF). [1,3,6-8,33,56-60]

Type: Associative fiber system

Anatomy: The arcuate fasciculus is a lateral associative bundle which interconnects primary and supplementary language-related areas in the frontal and temporal lobes. It is composed of long and short fibers connecting the perisilvian

cortex of the frontal, parietal, and temporal lobes. The fronto-parietal portion of the arcuate fasciculus encompasses a group of fibers with antero-posterior direction running lateral to the projection fibers of the corona radiata. These fibers start from the ventral premotor cortex, cross the projection fibers descending from the central sulcus and at the temporo-parietal junction the arcuate fibers arch around the lateral Sylvian fissure to continue downwards into the stem of the temporal lobe. They end within the white matter of the superior middle and inferior temporal gyrus. It is divided in an inner part which represent the primary language pathway (perisilvian between Broca's area and Wernicke´s area) and in an outer part which support the connections between supplementary language areas Dorsolateral prefrontal cortex, pars triangularis with middle and inferior temporal gyrus.

Brain Grid: Anterior terminations A1-A2-A3-A4, C1-C2, S2; Stem: A1-A2-A3-A4, C1-C2, S2 and A1-A4, C2, S3; Temporal terminations: A1-A4, C2-C3, S2-S3.

Functions: The AF is related (as primary language pathway) to linear encoding and articulation and speech recognition or phonologic decoding of heard speech. The outer supplementary pathway would be implicated in word selection, semantic aspects of language processing, and lexical–semantic processing.

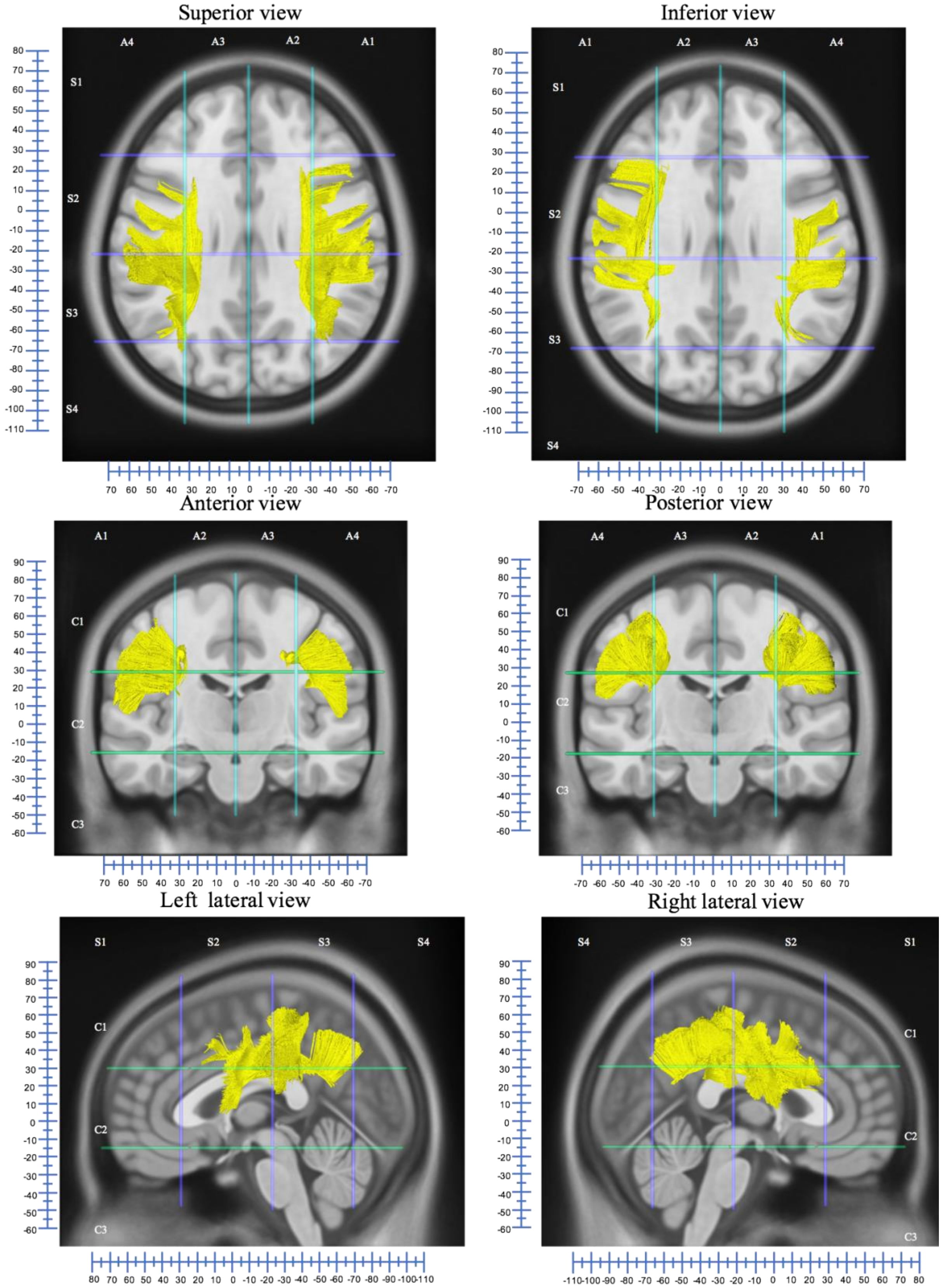

Figure 22. Horizontal segment of Superior Longitudinal Fasciculus (hSLF). [1,3,6,8,33,59,61-64]

Type: Associative fiber system

Anatomy: The hSLF connects temporo-parietal junction area and parietal lobe with the frontal lobe. It seems to include two different segments: from angular gyrus to caudal middle frontal gyrus and dorsal precentral gyrus representing the dorsal SLF, and from supramarginal gyrus to ventral precentral gyrus and pars opercularis to form the ventral SLF.

Brain grid: A1-A4, C1-C2, S2-S3

Functions: The hSLF integrates motor behavior and working memory, transferring somatosensory information, and contributing to the cortico-cerebellar system.

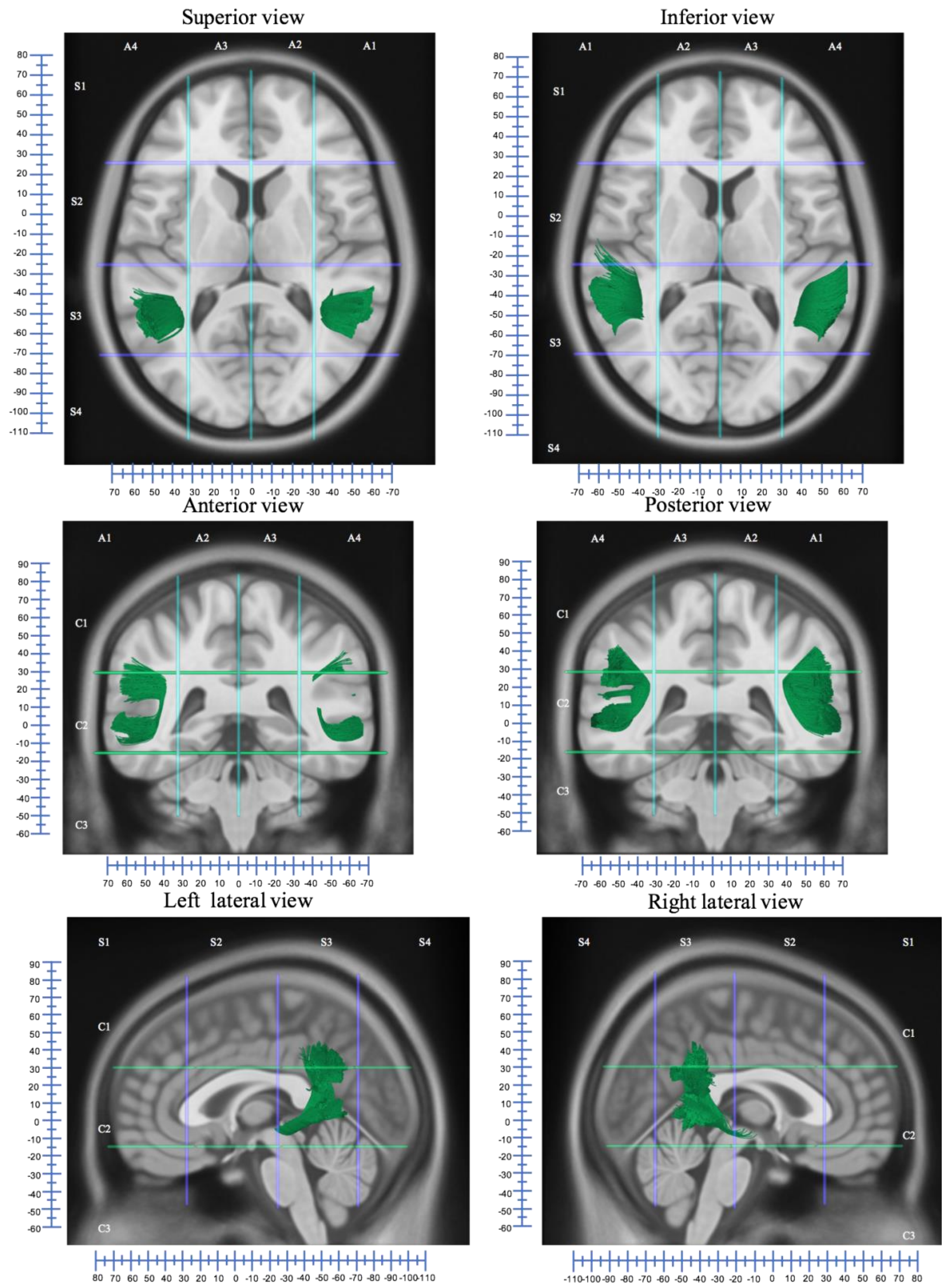

Figure 23. Vertical segment of Superior Longitudinal Fasciculus (vSLF or SLF TP). [1,3,6-8,33,51,59,61,63-66]

Type: Associative fiber system

Anatomy: The vSLF connects the temporal and parietal lobes together with fibers which are craniocaudally oriented from the posterior part of the superior temporal gyrus adjacent to the origin of the arcuate fasciculus, cranially to the superior parietal lobule. The SLF TP arises from the posterior portion of the superior parietal gyrus adjacent to the

origination of the arcuate fasciculus. As they ascend within the core of the temporo-parietal white matter, some of the fibers of the SLF TP insert into the inferior parietal lobe and the rest of the fibers ascend medially to the SLF II fibers and lateral to the superior parietal lobule connection of the middle longitudinal fasciculus fibers and insert into the superior parietal lobule.

Brain grid: Parietal terminations: A1-A4, C1-C2, S3; temporal terminations: A1-A4, C2, S3.

Functions: The vSLF integrates the access and retrieval of semantic information for language elaboration. Pure anomia has been associated with disruption or damage of this bundle.

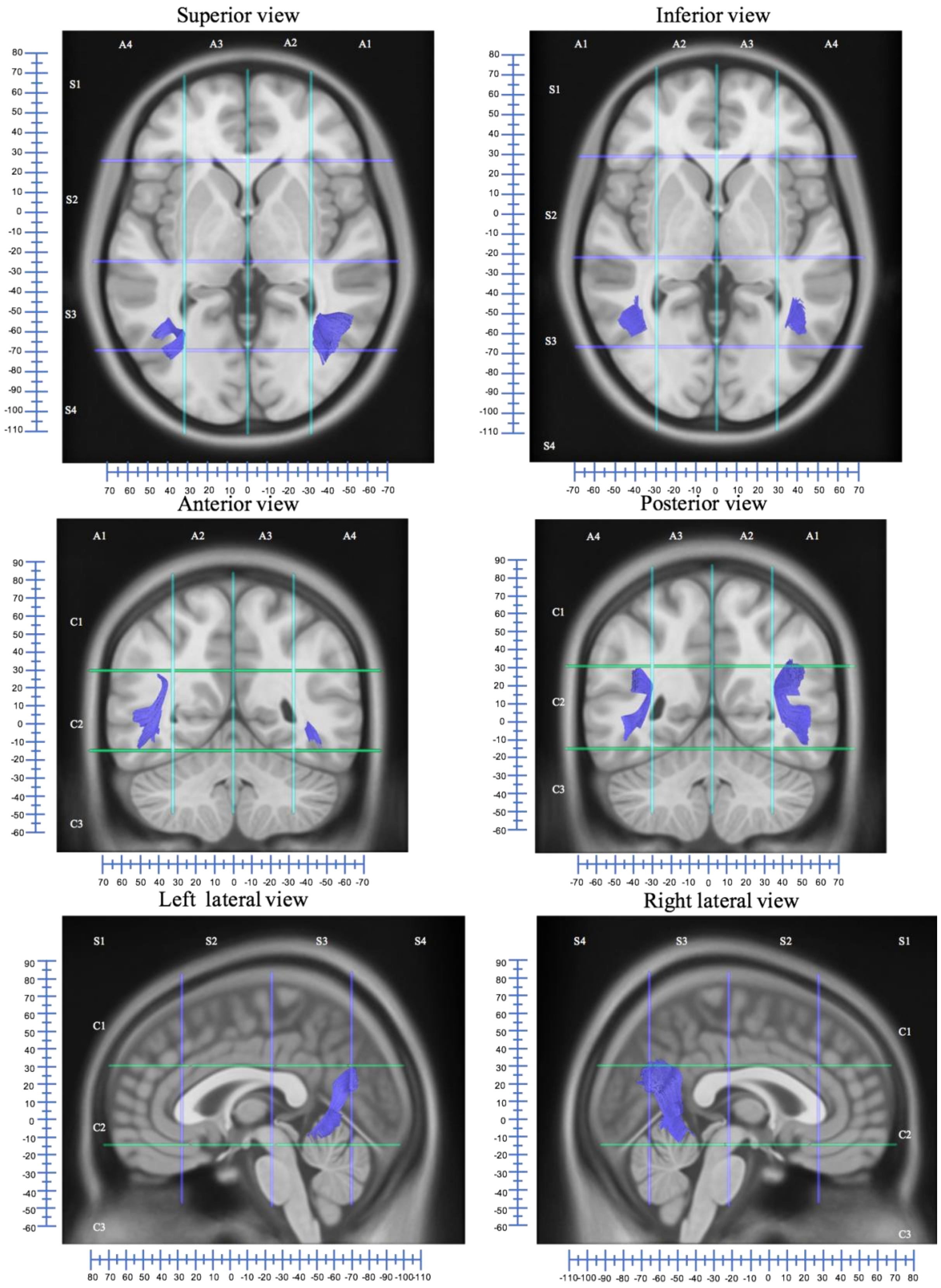

Figure 24. Vertical Occipital Fasciculus (VOF). [33,67,68-70]

Type: Associative fiber system

Anatomy: The vertical occipital fasciculus is the major fiber bundle connecting dorsolateral and ventrolateral visual cortex. VOF fibers ascend from the fusiform gyrus, immediately lateral to ILF, before branching laterally toward its cortical endpoints in the lateral occipital and inferior parietal lobes.

Brain Grid: A1-A4, C2, S3.

Functions: VOF is likely to integrate the communication of signals between regions on the ventral surface that are important for the perception of visual categories (e.g., words, faces, bodies, etc.) and regions on the dorsal surface involved in the control of eye movements, attention, and motion perception.

References.

1. Catani M. and Thiebaut de Schotten M. A diffusion tensor imaging tractography atlas for virtual in vivo dissections, Cortex, 2008; vol. 44, no. 8, pp. 1105–1132.
2. Mori S, Wakana S, van Zijl PCM, Poetscher N. MRI Atlas of Human White Matter, First Edition. London: Elsevier Science. 2005.
3. Wakana S, Caprihan A, Panzenboeck MM, Fallon JH, Perry M, Gollub RL, et al. Reproducibility of quantitative tractography methods applied to cerebral white matter. Neuroimage. 2007; 36(3):630–44. doi:10.1016/j.neuroimage.2007.02.049
4. Schmahmann J, Smith E, Eichler F, Filley CM. Cerebral White Matter: Neuroanatomy, Clinical Neurology, and Neurobehavioral Correlates. Ann N Y Acad Sci. 2008; 1142: 266–309, doi:10.1196/annals.1444.017.
5. Catani M, Howard RJ, Pajevic S, and Jones DK. Virtual in vivo interactive dissection of white matter fasciculi in the human brain. Neuroimage, 2002; 17: 77–94.
6. Catani M, Dell'acqua F, Bizzi A, Forkel SJ, Williams SC, Simmons A, et al. Beyond cortical localization in clinico-anatomical correlation. Cortex. 2012; 48(10):1262-87. doi: 10.1016/j.cortex.2012.07.001
7. Fernández-Miranda JC, Rhoton AL Jr, Alvarez-Linera J, Kakizawa Y, Choi C, de Oliveira EP. Three-dimensional microsurgical and tractographic anatomy of the white matter of the human brain. Neurosurgery. 2008; 62 (6 Suppl 3):989-1026; discussion 1026-8. doi: 10.1227/01.neu.0000333767.05328.49.
8. Fernandez-Miranda JC, Pathak S, Engh J, Jarbo K, Verstynen T, Yeh FC, et al. High-definition fiber tractography of the human brain: neuroanatomical validation and neurosurgical applications. Neurosurgery. 2012; 71:430–453.
9. Güngör A, Baydin S, Middlebrooks EH, Tanriover N, Isler C, Rhoton AL Jr. The white matter tracts of the cerebrum in ventricular surgery and hydrocephalus. J Neurosurg. 2017; 126(3):945-971.
10. Peltier J, Verclytte S, Delmaire C, Pruvo JP, Havet E, Le Gars D. Microsurgical anatomy of the anterior commissure: correlations with diffusion tensor imaging fiber tracking and clinical relevance. Neurosurgery. 2011; 69(2 Suppl Operative): ons241-6.
11. Wilde EA, Bigler ED, Haider JM, Chu Z, Levin HS, Li X, et al. Vulnerability of the anterior commissure in moderate to severe pediatric traumatic brain injury. J Child Neurol 21(9):769-776, 2006
12. Berlucchi G. Frontal callosal disconnection syndromes. 2012; Cortex. 2;48(1):36- 45.
13. De Benedictis A, Petit L, Descoteaux M, Marras CE, Barbareschi M, Corsini F, et al. New insights in the homotopic and heterotopic connectivity of the frontal portion of the human corpus callosum revealed by microdissection and diffusion tractography. Hum Brain Mapp. 2016; 37(12):4718-4735. doi: 10.1002/hbm.23339
14. Glickstein M. and Berlucchi G. Classical disconnection studies of the corpus callosum. Cortex, 2008; 44: 914–927.
15. Hofer S, Frahm J. Topography of the human corpus callosum revisited— comprehensive fiber tractography using diffusion tensor magnetic resonance imaging. NeuroImage. 2006; 32(3):989-994.
16. Schulte T, Müller-Oehring EM. Contribution of callosal connections to the interhemispheric integration of visuomotor and cognitive processes. Neuropsychol Rev. 2010; 20(2):174-190.

17. Van der Knaap LJ, van der Ham IJM. How does the corpus callosum mediate interhemispheric transfer? A review. Behav Brain Res. 2011; 223(1):211-221.

18. Gaffan D. and Wilson CRE. Medial temporal and prefrontal function: Recent behavioural disconnection studies in the macaque monkey. Cortex, 2008; 44: 928–935.

19. Ross ED. Sensory-specific amnesia and hypoemotionality in humans and monkeys: Gateway for developing a hodology of memory. Cortex, 2008; 44: 1010–1022.

20. Jeanmonod D, Schulman J, Ramirez R, et al. Neuropsychiatric thalamocortical dysrhythmia: surgical implications. Neurosurg Clin N Am, 2003; 14(2):251-65.

21. Jiang S, Luo C, Gong J, Peng R, Ma S, Tan S, et al. Aberrant Thalamocortical Connectivity in Juvenile Myoclonic Epilepsy. International Journal of Neural Systems, 2018; Vol. 27, No. 0 1750034. DOI: 10.1142/S0129065717500344

22. Ribary U. Dynamics of thalamo-cortical network oscillations and human perception. Progress in Brain Research, S. Laureys (Ed.). 2005; Vol. 150, pp127-142. ISSN 0079-6123. DOI: 10.1016/S0079-6123(05)50010-4

23. Crosby EC, Humphrey T, and Lauer EW. Correlative Anatomy of the Nervous System. Macmillian Co., New York. 1962.

24. Schmahmann J. and Pandya DN. Disconnection syndromes of basal ganglia, thalamus, and cerebrocerebellar systems. Cortex, 2008; 44: 1037–1066,

25. Alvarez, I, Schwarzkopf DS, Clark CA. Extrastriate projections in human optic radiation revealed by fMRI-informed tractography. Brain Struct Funct. 2015; 220(5):2519-32. doi: 10.1007/s00429-014-0799-4.

26. Welniarz Q, Dusart I and Roze E. The corticospinal tract: Evolution, development, and human disorders. Devel Neurobio, 2017; 77: 810–829. doi:10.1002/dneu.22455

27. Rudrauf D, Mehta S, and Grabowski T. Disconnection's renaissance takes shape: Formal incorporation in group-level lesion studies. Cortex, 2008; 44: 1084–1096.

28. Catani M. and Mesulam M. The arcuate fasciculus and the disconnection theme in language and aphasia: history and current state. Cortex, 2008; 44(8):953-61. doi: 10.1016/j.cortex.2008.04.002

29. Doricchi F, Thiebaut de Schotten M, Tomaiuolo F, Bartolomeo P. White matter (dis)connections and gray matter (dys)functions in visual neglect: Gaining insights into the brain networks of spatial awareness. Cortex, 2008; 44: 983–995

30. Epelbaum S, Pinel P, Gaillard R, Delmaire C, Perrin M, Dupont S, et al. Pure alexia as a disconnection syndrome: New diffusion imaging evidence for an old concept. Cortex, 2008; 44: 962– 974

31. Fox CJ, Iaria G, and Barton JJS. Disconnection in prosopagnosia and face processing. Cortex, 2008; 44: 996–1009.

32. Martino J, Vergani F, Robles SG, Duffau H. New insights into the anatomic dissection of the temporal stem with special emphasis on the inferior fronto-occipital fasciculus: implications in surgical approach to left mesiotemporal and temporoinsular structures. Neurosurgery. 2010; 66(3 Suppl Operative):4-12. doi: 10.1227/01.NEU.0000348564.28415.FA.

33. Martino J, De Lucas EM. Subcortical anatomy of the lateral association fascicles of the brain: A review. Clin Anat. 2014; 27(4):563-9. doi: 10.1002/ca.22321.

34. Panesar SS, Yeh FC, Deibert CP, Fernandes-Cabral D, Rowthu V, Celtikci, et al. A diffusion spectrum imaging-based tractographic study into the anatomical subdivision and cortical connectivity of the ventral external capsule: uncinate and inferior fronto-occipital fascicles. Neuroradiology. 2017; 59(10):971-987; doi: 10.1007/s00234-017-1874-3.

35. Sarubbo S, De Benedictis A, Maldonado IL, Basso G, Duffau H. Frontal terminations for the inferior fronto-occipital fascicle: anatomical dissection, DTI study and functional considerations on a multi-component bundle. Brain Struct Funct. 2013; 218(1):21-37. doi: 10.1007/s00429-011-0372-3.

36. Hau J, Sarubbo S, Perchey G, Crivello F, Zago L, Mellet E, et al. Cortical terminations of the inferior fronto-occipital and uncinate fasciculi: anatomical stem-based virtual dissection. Front Neuroanat. 2016; May 24;10:58,. doi: 10.3389/fnana.2016.00058

37. VonDer Heide RJ, Skipper LM, Klobusicky E, Olson IR. Dissecting the uncinate fasciculus: disorders, controversies and a hypothesis. Brain. 2013; 136(Pt 6):1692– 1707.

38. Alario FX, Chainay H, Lehericy S, and Cohen L. The role of the supplementary motor area (SMA) in word production. Brain Research, 2006; 1076(1): 129e143.

39. Boecker H, Dagher A, Ceballos-Baumann AO, Passingham RE, Samuel M, Friston KJ, et al. Role of the human rostral supplementary motor area and the basal ganglia in motor sequence control: Investigations with H2 15O PET. Journal of Neurophysiology, 1998; 79(2): 1070e1080.

40. Catani M, Dell'Acqua F, Vergani F, Malik F, Hodge H, Roy P, et al. Short frontal lobe connections of the human brain. Cortex, 2012; 48(2):273-91. doi: 10.1016/j.cortex.2011.12.001.

41. Serra L, Gabrielli GB, Tuzzi E, Spanò B, Giulietti G, Failoni V, et al. Damage to the Frontal Aslant Tract Accounts for Visuo-Constructive Deficits in Alzheimer's Disease. J Alzheimers Dis, 2017; 60(3):1015-1024. doi: 10.3233/JAD-170638.

42. Makris N, Papadimitriou GM, Kaiser JR, Sorg S, Kennedy DN, Pandya DN. Delineation of the middle longitudinal fascicle in humans: A quantitative, in vivo, DT-MRI study. Cereb Cortex. 2009; 19: 777–785

43. Makris N, Preti M G, Asami T, Pelavin P, Campbell B, Papadimitriou GM, et al. Human middle longitudinal fascicle: variations in patterns of anatomical connections. Brain Structure & Function, 2013; 218(4), 951–968. doi:10.1007/s00429-012- 0441-2.

44. Makris N, Preti MG, Wassermann D, Rathi Y, Papadimitriou GM, Yergatian C, et al. Human middle longitudinal fascicle: segregation and behavioral-clinical implications of two distinct fiber connections linking temporal pole and superior temporal gyrus with the angular gyrus or superior parietal lobule using multi-tensor tractography. Brain Imaging and Behavior, 2013; 7(3), 335–352. doi:10.1007/s11682-013-9235-2.

45. Makris N, Zhu A, Papadimitriou GM, Mouradian P, Ng I, Scaccianoce E, et al. Mapping temporo-parietal and temporo-occipital cortico-cortical connections of the human middle longitudinal fascicle in subject-specific, probabilistic, and stereotaxic Talairach spaces. Brain Imaging Behav. 2017; 11(5):1258-1277. doi: 10.1007/s11682-016-9589-3.

46. Maldonado IL, de Champfleur NM, Velut S, Destrieux C, Zemmoura I, Duffau H. Evidence of a middle longitudinal fasciculus in the human brain from fiber dissection. J Anat, 2013; 223(1), 38–45, doi:10.1111/joa.12055.

47. Martino J, da Silva-Freitas R, Caballero H, Marco de Lucas E, García-Porrero JA, Vázquez-Barquero A. Fiber dissection and diffusion tensor imaging tractography study of the temporoparietal fiber intersection area. Neurosurgery, 2013; 72(1 Suppl Operative), 87–97 discussion 97–88; doi:10.1227/NEU.0b013 e318274294b.

48. Menjot de Champfleur N, Lima Maldonado I, Moritz-Gasser S, Machi P, Le Bars E, Bonafé A, et al. Middle longitudinal fasciculus delineation within language pathways: a diffusion tensor imaging study in human. European Journal of Radiology, 2013; 82(1), 151–157. doi:10.1016/j.ejrad.2012.05.034.

49. Wang Y, Fernandez-Miranda JC, Verstynen T, Pathak S, Schneider W, Yeh FC. Rethinking the role of the middle longitudinal fascicle in language and auditory pathways. Cereb Cortex, 2013; 23(10), 2347–2356,. doi:10.1093/cercor/bhs225.

50. Catani M, Jones DK, Donato R, ffytche DH. Occipito-temporal connections in the human brain. Brain. 2003; 126:2093–2107.

51. Catani M, Jones DK, ffytche DH. Perisylvian language networks of the human brain. Ann Neurol. 2005; 57(1):8-16.

52. Ffytche DH and Catani M. Beyond localization: from hodology to function. Philosophical Transactions of the Royal Society of London B Biological Sciences, 2005; 360: 767–779.

53. Ffytche DH. The hodology of hallucinations. Cortex, 2008; 44: 1067–1083.

54. Latini F. New insights in the limbic modulation of visual inputs: the role of the inferior longitudinal fasciculus and the Li-Am bundle. Neurosurg Rev. 2015; 38(1):179-89; discussion 189-90. doi: 10.1007/s10143-014-0583-1.

55. Latini F, Mårtensson J, Larsson EM, Fredrikson M, Åhs F, Hjortberg M et al. Segmentation of the inferior longitudinal fasciculus in the human brain: A white matter dissection and diffusion tensor tractography study. Brain Res. 2017; 15;1675:102-115. doi: 10.1016/j.brainres.2017.09.005.

56. Amunts K, Schleicher A, Zilles K. Outstanding language competence and cytoarchitecture in Broca’s speech region. Brain Lang. 2004; 89:346–353.

57. Fernandez-Miranda JC, Wang Y, Pathak S, Stefaneau L, Verstynen T, Yeh FC. Asymmetry, connectivity, and segmentation of the arcuate fascicle in the human brain. Brain Struct Funct. 2015; 220(3):1665-80. doi: 10.1007/s00429-014-0751-7

58. Hickok G, Poeppel D. Dorsal and ventral streams: a framework for understanding aspects of the functional anatomy of language. Cognition. 2004; 92:67–99.

59. Makris N, Kennedy DN, McInerney S, et al. Segmentation of subcomponents within the superior longitudinal fascicle in humans: a quantitative, in vivo, DT-MRI study. Cereb Cortex. 2005; 15:854–869.

60. Vandenberghe R, Price C, Wise R, Josephs O, Frackowiak RS. Functional anatomy of a common semantic system for words and pictures. Nature. 1996; 383:254–256.

61. Dick AS, Tremblay P. Beyond the arcuate fasciculus: consensus and controversy in the connectional anatomy of language. Brain, 2012; 135(Pt 12):3529-50. doi: 10.1093/brain/aws222.

62. Martino J, De Witt Hamer PC, Berger MS, et al. Analysis of the subcomponents and cortical terminations of the perisilvian superior longitudinal fasciculus: a fiber dissection and DTI tractography study. Brain Struct Funct. 2013; 218(1):105-21. doi: 10.1007/s00429-012-0386-5.

63. Ramnani N. Frontal lobe and posterior parietal contributions to the cortico-cerebellar system. Cerebellum, 2012; 11(2):366–383. doi:10.1007/s12311-011-0272-3

64. Wang X, Pathak S, Stefaneanu L, Yeh FC4, Li S2, Fernandez-Miranda JC. Subcomponents and connectivity of the superior longitudinal fasciculus in the human brain. Brain Struct Funct. 2016; 221(4):2075-92. doi:10.1007/s00429-015-1028-5.

65. Kamali A, Flanders AE, Brody J, Hunter JV, Hasan KM. Tracing superior longitudinal fasciculus connectivity in the human brain using high resolution diffusion tensor tractography. Brain Struct Funct. 2014; 219(1):269-81. doi: 10.1007/s00429-012-0498-y.

66. Wu Y, Sun D, Wang Y, Wang Y, Wang Y. Tracing short connections of the temporo-parieto-occipital region in the human brain using diffusion spectrum imaging and fiber dissection. Brain Res, 2016; 1646:152-159. doi: 10.1016/j.brainres.2016.05.046

67. Keser Z, Ucisik-Keser FE, Hasan KM. Quantitative Mapping of Human Brain Vertical-Occipital Fasciculus. J Neuroimaging. 2016; 26(2):188-93. doi: 10.1111/jon.12268

68. Takemura H, Rokem A, Winawer J, Yeatman JD, Wandell BA, Pestilli F. A major human white matter pathway between dorsal and ventral visual cortex. Cereb Cortex. 2016; 26(5):2205-2214. doi: 10.1093/cercor/bhv064

69. Weiner KS, Yeatman JD, Wandell BA. The posterior arcuate fasciculus and the vertical occipital fasciculus. Cortex. 2016; pii: S0010-9452(16)30050-8. doi: 10.1016/j.cortex.2016.03.012

70. Yeatman JD, Weiner KS, Pestilli F, Rokem A, Mezer A, Wandell BA, et al. The vertical occipital fasciculus: a century of controversy resolved by in vivo measurements. Proc Natl Acad Sci USA. 2014; 111:E5214-23.
